# BKDRP: a biological knowledge-driven approach for drug response prediction using multi-omics data in cancer cell lines

**DOI:** 10.1186/s12859-026-06406-2

**Published:** 2026-03-17

**Authors:** Koyel Mandal, Sanghamitra Bandyopadhyay

**Affiliations:** Machine Intelligence Unit, Indian Statistical Institute, Kolkata, West Bengal 700108 India

**Keywords:** Drug response prediction, Multi-omics data, Cancer cell line, Deep neural network, Morgan fingerprint, Biological knowledge

## Abstract

****Background**:**

Cancer heterogeneity results in patients with the same diagnosis responding differently to drugs, making treatments extremely challenging. Advances in computational power enable personalized treatments that suppress tumors and extend patient survival. Therefore, accurate prediction of cancer cell response to a particular medication is of utmost importance. Current deep learning-based models have achieved impressive accuracy, but they often function as a “black box” and cannot explain the reason for the prediction. To address this limitation, we develop a deep learning-based model, BKDRP, which incorporates prior biological information into the architecture, along with molecular fingerprints of drugs, while embedding biological priors into its architecture. Specifically, it incorporates the fact that genes encode proteins that combine to form protein complexes, which in turn regulate biological pathways, ultimately targeted by drugs.

****Results**:**

We evaluate BKDRP on the GDSC (Genomics of Drug Sensitivity and Cancer) cell line dataset using multi-omics gene expression, protein expression, mutation, and copy number variation. Four rigorous experiments have been conducted to test the model’s generalizability: prediction of unknown drug–cell line responses, responses to unseen drugs (LODO: Leave-One-Drug-Out), responses to unseen cell lines (LOCLO: Leave-On-Cell-Line-Out), and responses across unseen cancer types (LOCO: Leave-One-Cancer-Out). The performance of the proposed method and baseline algorithms is assessed using two metrics: Area Under the ROC Curve (AUC) and Area Under the Precision-Recall Curve (AUPR). The experimental results demonstrate that BKDRP performs well in different evaluation techniques. Notably, BKDRP has achieved an AUC of 0.8845, surpassing traditional machine learning and deep learning approaches and demonstrating robustness in handling biological variability across cancer types. A case study of lung adenocarcinoma (LUAD) highlights known biomarkers (*KRAS*, *EGFR*, *STK11*), key proteins (SOCS1, HSPA8, SMC3), and drugs (Erlotinib, Palbociclib) that are consistent with the literature.

****Conclusions**:**

In conclusion, BKDRP presents a novel biological knowledge-driven deep neural network model for cancer drug response prediction that shows strong predictive accuracy and interpretability. By integrating multi-omics data and incorporating domain knowledge, BKDRP has the strong potential for applications in biomarker discovery and the advancement of personalized oncology.

## Background

Cancer, a highly complex disease, is one of the primary causes of morbidity and mortality worldwide. Fortunately, from the year 2015 to 2019, there was a notable decrease in death rates across all demographic groups by 2.3% per year for men and by 1.9% per year for women [1]. Early detection of cancer and effective management in treatment procedures have helped in reducing death rates for 11 of the 19 most common cancers in men and 14 of the 20 most common cancers in women [1]. Despite the overall improvement in prognosis, the response to cancer treatment remains unpredictable. The molecular analysis of cancer reveals that cancer originating in different organs may exhibit similar features, whereas cancer from the same organ can be distinct [2]. The heterogeneous nature of cancer results in patients with the same diagnosis responding differently to anticancer drugs, making treatment challenging and extremely difficult to manage [3]. The diversity in drug responses is believed to be due to complex and distinct genomic instability [2]. This variability necessitates personalized approaches to treatment, emphasizing the importance of precision medicine. Nowadays, with the significant advancement in high-throughput technology, precision medicine has been gaining popularity for future medical science by exploring new cancer treatment methods based on the genomic profile of each patient [4]. The main characteristic of precision oncology is that it provides treatments tailored to the specific nature of malignant tumors. Developing and accessing targeted therapies requires the lengthy and costly process of drug development and evaluation through clinical trials, which are the most direct methods for assessing drug efficacy and toxicity [5]. One of the major solutions to this problem is to develop genomic predictors of drug response using large panels of cancer cell lines. In contrast, computational methods offer a cheaper, faster, and scalable alternative.

The earliest anti-cancer drug screens in human tumor cell lines database are the National Cancer Institute 60 (NCI60) [6]. Genomics of Drug Sensitivity in Cancer (GDSC) [7] and Cancer Cell Line Encyclopedia (CCLE) [8] have become two major studies for cancer cell lines. These projects generate an enormous amount of drug response data for about 250 drugs across 1000 cancer cell lines. Additionally, these datasets are integrated with genomic data for the cultured cancer cell lines that play a significant role in studying drug response prediction (DRP). Although cancer cell lines are very different from the original tissue or cancer samples, they remain a valuable resource in finding new anti-cancer drugs in cancer biology [9]. In the field of precision medicine, cancer cell lines have contributed to exploring the molecular mechanisms of cancer treatment and discovering novel anti-cancer therapies [4]. Therefore, cancer cell line DRP can be helpful to improve treatment plans, treatment effects, and treatment costs [10–12].

Cancer DRP algorithms are categorized into two groups: classification-based algorithms, i.e., predicting the sensitivity of drug-cell line pairs, and regression-based methods, i.e., estimating quantitative drug response values such as the half-maximal inhibitory concentration (IC_50_) [13]. Many studies have attempted to incorporate various types of molecular data, including DNA copy number variation, mutations, gene expression data, methylation, and proteomics, to achieve better accuracy in DRP. Early approaches applied machine learning approaches such as support vector machines [5, 14], random forests, and elastic-net regression. Recent advances have shifted from traditional machine learning to deep learning, which can automatically learn feature representations from high-dimensional molecular data [15, 16]. Deep neural networks—including feedforward networks, convolutional neural networks (CNNs), and recurrent architectures—have demonstrated reasonably good performance in capturing non-linear relationships between genomic features and drug responses without extensive feature engineering [17, 18].

A plethora of research work has been proposed in this area [15, 19–22]. An integrative framework, named Predict Drug Response in Cancer Cells (PDRCC), is proposed by Wang et al. [14] for predicting chemotherapeutic responses in cancer patients using cancer genomic alterations, compound chemical properties, and therapeutic characteristics. Wang et al. [22] have developed a similarity-regularized matrix factorization (SRMF) method for predicting drug responses, based on the principle that cell lines and drugs with similar chemical properties exhibit similar responses. This method also identified novel drug-cancer gene associations. Zhang et al. [23] have proposed a novel heterogeneous network-based method for drug response prediction (HNMDRP) to efficiently predict cell line drug associations by utilizing the genomic profile of the cell line, protein-protein interaction (PPI), drug target, and drug-chemical structure.

More recently, the research work for drug response prediction has largely adopted advanced machine learning methods, such as deep neural networks (DNN) [18, 24–27]. Menden et al. [28] have proposed a machine learning approach using feed-forward neural networks and random forests to predict cancer cell sensitivity to drugs based on genomic and chemical features. In the study by Liu et al. [18], a twin Convolutional Neural Network (tCNNS) for drugs in Simplified Molecular-Input Line-Entry System (SMILES) is proposed to predict cancer drug response. tCNNS introduces a dual-branch architecture that uses separate convolutional networks to extract features from drug SMILES representations and cancer cell line genetic profiles, followed by a fully connected network for interaction prediction. DeepCDR [27], a hybrid graph convolutional network, has been developed by Liu et al. The model consists of a Uniform Graph Convolutional Network (UGCN) and multiple subnetworks that automatically learn latent representations of topological structures among atoms and bonds of drugs. Choi et al. [15] have proposed a novel Reference Drug-based Neural Network (RefDNN) that leverages a set of reference drugs to learn representations for both high-dimensional gene expression vectors and the molecular structure vectors of drugs for effective DRP, such as resistance or sensitivity. The work is also based on the assumption that similar chemicals tend to have similar effects.

The majority of the proposed work in DRP does not use prior biological knowledge during model development. Few works have incorporated the domain knowledge. Snow et al. [29] have developed BDKANN, an interpretable deep neural network that incorporates biological pathway knowledge to predict cancer drug response from cell line gene expression data, demonstrating meaningful network interpretations for understanding drug action. However, BDKANN is limited to gene expression only, which may not capture the comprehensive cellular states that multi-omics integration can provide for more robust and accurate drug response prediction. A pathway-guided DNN that constrains connections between gene and pathway layers based on KEGG pathway knowledge to predict drug sensitivity in cancer cells is presented in the article [30].

Another interpretable model for predicting anticancer drug response is DRPreter (drug response predictor and interpreter) [31]. The model utilizes knowledge-guided graph neural networks to divide cell-line graphs into pathway-specific subgraphs and incorporates a transformer-based module to identify drug-pathway relationships for enhanced interpretability. Xie et al. [32] have recently introduced DrugVNN, an end-to-end interpretable framework for predicting drug responses. This framework integrates knowledge-guided visible neural networks (VNN) to extract gene features from cell lines and employs a node-edge communicative message passing network (CMPNN) to learn comprehensive drug representations. Lao et al. [26] have proposed DeepAEG, an end-to-end deep learning model that uses a hybrid graph convolutional network with edge update mechanisms to predict IC_50_ values from drug molecular structures and multi-omics cancer cell data.

Although existing interpretable models have made remarkable progress, they still suffer from several critical limitations. Models like BDKANN and PathDNN depend solely on gene expression data and ignore the integration of multi-omics data for capturing a comprehensive molecular state. On the other side, advanced models like DeepAEG and DeepCDR incorporate multi-omics data but primarily focus on molecular structure learning without adequately modelling how drugs specifically interact with biological pathways and protein networks. To fill this research gap, we have developed a model that integrates multi-omics data through a protein-pathway hierarchical architecture while incorporating drug fingerprint information for enhanced molecular representation.

Despite having tremendous progress in DRP models, many of these methods function as “black boxes”, and the explanation behind these models is unknown. The lack of transparency is particularly concerning in cancer therapy because it is important to know the explanation of why a drug is expected to work for a particular patient. Current models often fail to provide explanations behind the model predictions. Additionally, most of the models use genes as features, ignoring the crucial fact that genes do not operate in isolation; rather, they work in biological networks or pathways. In the context of DRP, genes encode proteins that combine together to form protein complexes, which in turn regulate different biological pathways. Eventually, pathways can be disrupted by a particular drug. Therefore, we are motivated to incorporate biological domain knowledge in the computational models that will definitely improve the accuracy of DRP. This allows us to trace back to specific nodes that represent drugs, pathways, proteins, or genes. Limited attempts have been made to include prior biological knowledge to predict drug responses.

To address this limitation, we have developed a novel method named the Biological Knowledge-Driven Drug Response Prediction (BKDRP) model for effective drug response prediction. BKDRP incorporates hierarchical biological information that models the gene $\rightarrow$ protein $\rightarrow$ pathway $\rightarrow$ drug information flow, enabling both accurate predictions and interpretability. The key contributions of this article are as follows: (i) a biologically-constrained neural network architecture, (ii) comprehensive multi-omics integration with weighted average, (iii) rigorous evaluation using five-fold cross-validation, Leave-One-Drug-Out (LODO), Leave-One-Cancer-Out (LOCO), and Leave-One-Cell-Line-Out (LOCLO), and (iv) a biological validation framework for model interpretability.

## Methods

**Fig. 1:**
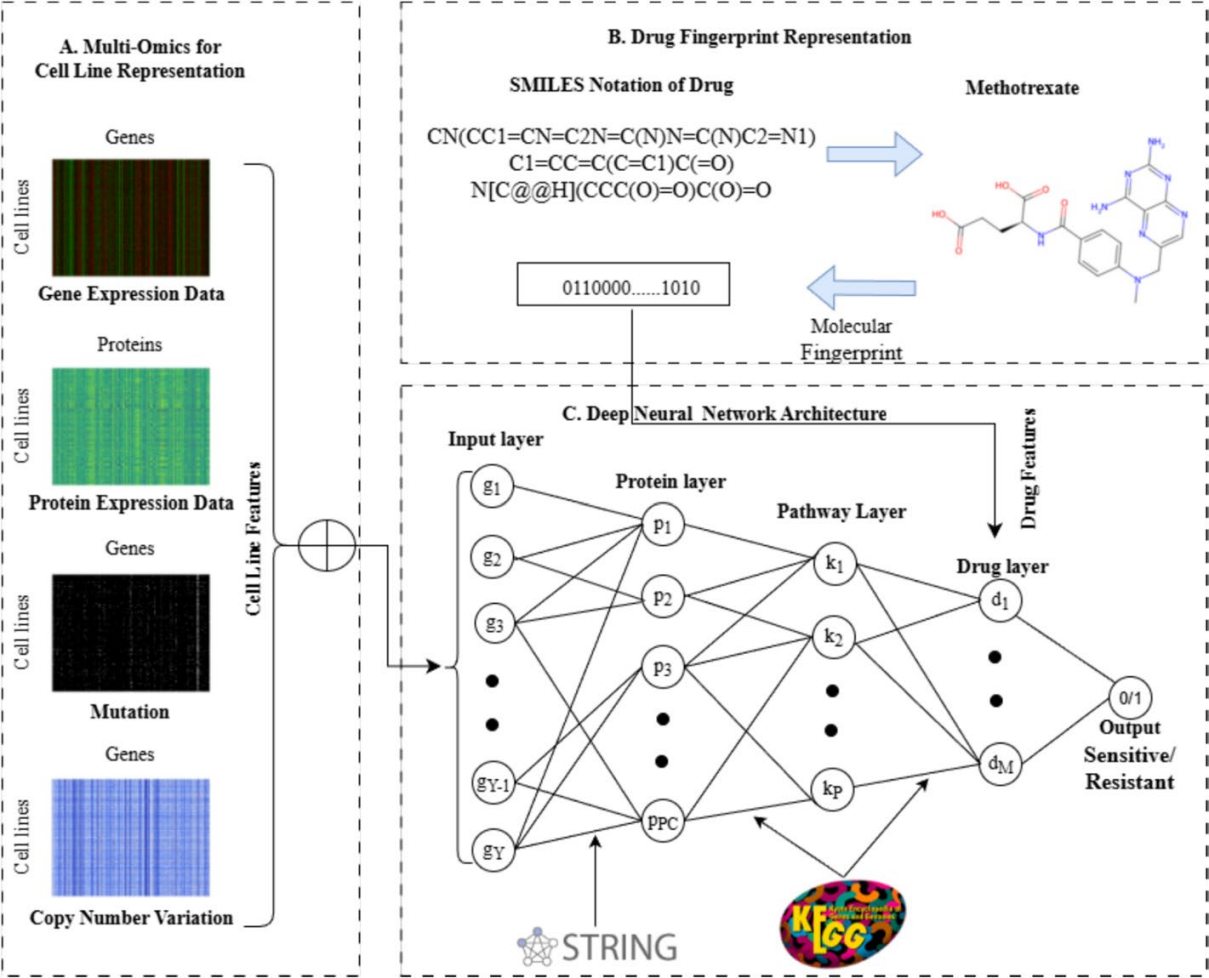
The overview of the Biological Knowledge-Driven Approach for Drug Response Prediction (BKDRP). **A** Multi-omics for cell line representation, integration of gene expression, protein expression, mutation, and copy number variation (CNV). **B** Drug fingerprint representation using SMILES notation and molecular fingerprints (example: Methotrexate). **C** Deep neural network architecture considers cell line features and drug features to predict drug sensitivity or resistance

In this section, we describe the proposed cancer DRP model, i.e., BKDRP, in detail. The methodology can be broadly divided into three components: (A) multi-omics for cell line representation, (B) drug fingerprint representation, and (C) a deep neural network architecture that predicts cancer cell line drug response using genomic and drug features. Each input node represents a single multi-omics feature (e.g., gene expression, mutation, CNV, or protein); a cell line is represented by the integration of all such feature nodes across the four omics types. BKDRP is a deep feedforward neural network with some constraints on the number of hidden layers, nodes, and edges. The constraints on hidden layers provide insights into the flow of biological information across layers. Similarly, limiting the number of edges allows us to use a larger number of nodes without increasing the number of edges, resulting in a smaller number of parameters compared with a fully connected network with the same number of nodes. Furthermore, restricting the number of nodes that do not contribute to the computation adds benefits; it acts as a biologically motivated dropout mechanism, which is more meaningful than random dropout. Furthermore, BKDRP reduces the effective parameter space due to its connectivity constraints compared to a fully connected network of similar depth. The workflow of this study is illustrated in Fig. 1.

Let $\mathcal {D}=\{d_1,d_2,\ldots , d_A\}$ be a set of *A* number of drugs, and $\mathcal {C}=\{c_1, c_2,\ldots , c_B\}$ be a set of *B* number of cancer cell lines. $\mathcal {R}(c_i,d_j)$ denotes the binary response of the cell line $c_i$ to the drug $d_j$. We obtain the IC$_{50}$ values from the GDSC dataset to do binary categorization of cancer cell lines and drug responses. We classify the IC$_{50}$ values using the maximum screening concentration of drug max_conc(*d*_*j*_), following the method described in the previously published work [9]. We define the task of DRP as a binary classification problem into two classes, as shown below:

1 $$\mathcal{R}(c_i,d_j)=\begin{cases}1, & {\mathrm{if}} \exp({\mathrm{IC}}_{50}(c_i,d_j)) \le \text{max{\_}conc}(d_j) \\0, & \mathrm{otherwise}\end{cases}$$

If $\mathcal {R}(c_i,d_j)=1$, it represents that the cell line $c_i$ is sensitive to the drug $d_j$; else, it is resistant. Mathematically, the problem can be defined as $\mathcal {R}(c_i,d_j) = \mathcal {F}(d_i,c_j)$, where $\mathcal {F}$ is BKDRP to predict the response $\mathcal {R}$ of cancer $c_i$ to the treatment by drug $d_j$.

### Multi-omics for cell line representation

The features of the cell lines are characterized by four types of different omics data, specifically gene expression, copy number variation, protein expression, and mutation data. We define the gene expression matrix $GE\in \mathbb {R}^{N\times Q}$ and the set of genes $\mathcal {G}=\{g_1,g_2,\ldots ,g_Q\}$, where *N* and *Q* are the total number of cell lines and the number of genes, respectively. The cell $ge(c_i, g_j)$ of the gene expression matrix captures the expression level of cell $c_i$ and gene $g_j$. The occurrence of multiple gene mutations is a key factor in cancer development [33]. Therefore, in this study, we incorporate mutation data to capture the genetic alterations associated with drug response. Specifically, we consider the Variant Allele Frequency (VAF) of mutations, which represents the proportion of sequencing reads that contain a variant at a given genetic locus [34]. In general, gene mutation involves numerous variations, such as deletions, insertions, splice site mutations, missense mutations, and nonsense mutations, among others. Mathematically, we represent mutation data as $Mut \in \mathbb {R}^{N \times R}$, where *R* is the total number of mutated genes. Each cell $mut(c_i,g_j)$ of the mutation data represents the VAF of cell $c_i$ and gene $g_j$. Another key feature of cell lines is the copy number variation (CNV). CNV represents the number of gene copies in a cell and refers to structurally variable regions where differences in copy numbers are observed between two or more genes. It can be described as $CNV \in \mathbb {R}^{N\times S}$, where *S* is the number of genes. Similarly, each cell of $cnv(c_i, g_j)$ describes the copy number variation of cell line $c_i$ and gene $g_j$. Next, we have taken into account the protein expression data consisting of *N* cell lines and *T* proteins. It is represented as $Protein \in \mathbb {R}^{N\times T}$, where $\mathcal {P}=\{p_1,p_2,\ldots ,p_T\}$ is the set of proteins, and each cell of the matrix defines the protein expression levels of cell line $c_i$ and protein $p_j$.

The feature representation of each cell line is defined by concatenating *Z* number of genomic features $\{g_1,g_2,\ldots , g_Q\}$ $\cup \{g_1,g_2,\ldots ,g_R\}$ $\cup \{g_1,g_2,\ldots ,g_S\}$ $\cup \{p_1,p_2,\ldots ,p_T\}$, where $Z=Q+R+S+T$. This facilitates the early integration of multi-omics data. It is noteworthy that all genes or proteins across different omics datasets are not essentially identical, as some genes may be present in multiple omics types, while others may appear in only one. To ensure consistent representation, when a gene is present in more than one omics, we compute its weighted average based on its availability across datasets. Let *Y* be the number of unique genes/proteins $\mathcal {G}_{unique} = \{g_1,g_2,\ldots , g_Y\}$ and $Y\le Z$. The integrated representation of cell line $c_i$ and gene $g_j$ is defined as $F(c_i,g_j)$, where $i=1,2,\ldots ,N$ represents the total *N* number of cell lines and $j=1,2,\ldots ,Y$ denotes the total *Y* number of genes.

2 $$\begin{aligned} F(c_i,g_j) = \frac{1}{|\mathcal {M}|}\sum _{\mathcal {M}\in \{GE, Mut, CNV, Protein\}} 1_{g_j\in \mathcal {M}}\times \mathcal {M}(c_i,g_j) \end{aligned}$$

here $1_{g_j\in \mathcal {M}}$ is an indicator that equals 1 if gene $g_j$ is present in omics dataset $\mathcal {M}$, and 0 otherwise.

### Drug fingerprint representation

SMILES is a chemical notation used to represent the structure of a drug molecule and is one of the most common and widely accepted formats in cheminformatics [18, 25, 35]. In this notation, each molecule is characterized by a string of symbols generated by a graph traversal algorithm. SMILES works as an intermediate step for other representations like descriptors, fingerprints, and graph structure. However, since the length of SMILES varies from one drug to another, a standardized representation is required to ensure uniformity in the number of features. To address this, fingerprints (FPs) are commonly used to describe molecular structures. FPs are the binary vector of fixed sizes, such as 512, 1024, or 2048, demonstrating the presence and absence of a particular substructure. Several cheminformatics packages implement multiple algorithms to generate fingerprints (FPS). One of the most widely used Python open-source packages is RDKit. In this study, we use Morgan fps, also known as Extended Connectivity Fingerprints (ECFPs), which are a type of circular fingerprint generated using the Morgan algorithm. ECFPs numerically encode the atomic neighborhoods within a molecule into binary representations.

### Deep neural network architecture

The neural network architecture is defined to reflect the biological hierarchy and relationships between layers. The model initiates with an input layer that receives integrated multi-omics features of each cell line, including gene expression, mutation, copy number variation, and protein expression. When multiple omics values exist for the same gene, their measurements are averaged to obtain a single value representation of that gene. Following the input layer, the network comprises three hidden layers: protein complex, pathway, and drug layers, and, finally, an output layer. Each node in the input layer corresponds to a gene having at least one interaction with the first hidden, i.e., protein complex layer, based on the protein–protein interaction (PPI) network. Similarly, the protein complex layer connects to the second hidden layer, named the pathway layer, using protein-pathway associations from Kyoto Encyclopedia of Genes and Genomes (KEGG), where an edge exists if a protein participates in that pathway. The last hidden layer presents the drug layer, which connects the pathway layer. The output layer performs binary classification to predict whether the response is sensitive or resistant. This connectivity between layers is implemented through adjacency matrices that act as masks over the weight matrices, ensuring that only biologically valid connections are trainable. Moreover, the model’s weights are learned during the training process. Additionally, the model is dynamically refined through a pruning mechanism that removes biologically irrelevant nodes and connections during construction. The entire process is described in detail next.

The first hidden layer in the model represents the *PC* number of protein complexes denoted as $\mathcal {P} = \{p_1,p_2,\ldots ,p_{PC}\}$ to which the genes are mapped. To establish the connection between genes and protein complexes, we have used the STRING v10.5 database [36], which provides comprehensive information on protein-protein interaction scores. These connections are supported only by domain expert knowledge, which ensures that the connections are biologically relevant. This layer significantly reduces the number of nodes, thereby optimizing memory usage in the model.

The second layer corresponds to pathways $\mathcal {K} = \{k_1,k_2,\ldots ,k_P\}$, where *P* is the total number of significant pathways. Each node is a pathway that a protein layer of the previous layer is part of. To know the significant pathways, we perform Gene Set Enrichment Analysis (GSEA) using the KEGG pathway database [37]. A pathway is said to be significant if its *p*-value is less than or equal to a significant cutoff, i.e., 5%. To achieve this, we use the GSEApy open-source Python package, which identifies pathways strongly associated with proteins. The combined enrichment score obtained from the analysis is used as the weight to reflect the gene-pathway relationships.

The third hidden layer is designed to represent a total of *M* nodes that correspond to drugs $\mathcal {D} =\{d_1,d_2,\ldots ,d_M\}$. The connections between drugs and their respective pathways in the preceding layer are defined by a binary matrix using the KEGG pathway database [37]. This matrix encodes 1 and 0 if a drug targets a specific pathway, indicating a connection or no connection, respectively. The fingerprint representation of each drug is concatenated in this layer, which ultimately contributes to predicting the final binary output.

The output of each layer in the model is calculated as delineated below.

3 $$\begin{aligned} y^{(l)} = f[(M^{(l)}*W^{(l)})^TX^{(l-1)}+b^{(l)}] \end{aligned}$$

Here *l*, *f*, *M*, *W*, *X*, *b*, and * are the layer, activation function, masked matrix, weight matrix, input matrix, bias vector, and Hadamard product, respectively. For each layer *l*, a weight matrix $W^{(l)}\in \mathbb {R}^{n_{l-1}\times n_l}$ and a binary mask matrix $M^{l}\in \{0,1\}^{n_{l-1}\times n_l}$ are associated with the proposed model, where $n_{l-1}$ and $n_l$ are the number of nodes in the previous and current layers, respectively. The mask matrix represents the biologically relevant connections between layers derived from biological databases. More specifically,

4 $$\begin{aligned} M_{ij}^{(l)} = {\left\{ \begin{array}{ll} 1, & \text {if node { i} in layer (l-1) is biologically connected to node { j} in layer { l}} \\ 0, & \text {Otherwise} \end{array}\right. } \end{aligned}$$

During the forward pass, the Hadamard operation ensures that only biologically valid weights are updated in the training process. In this way, $W^{(l)}$ contains the trainable parameters that correspond to known biological interactions, while the masked-out connection $M_{ij}^{(l)}=0$ is excluded from the gradient updates.

In the third hidden layer, the output ($y_{drug}$) is concatenated with the drug fingerprints ($X_{fingerprints}$) to form a combined representation, $y_{concat} = y_{drug} \oplus X_{fingerprints}$, where $\oplus$ is a concatenation operation. The activation function used in each hidden layer is Rectified Linear Unit (ReLU), which is defined below.

5 $$\begin{aligned} f(x) = {\left\{ \begin{array}{ll} x, & \text {if } x > 0 \\ 0, & \text {if } x \le 0 \end{array}\right. } \end{aligned}$$

The output layer computes the final prediction as follows.

6 $$\begin{aligned} y = \sigma (y_{concat}\cdot W + b) \end{aligned}$$

The activation function in the output layer is calculated by the sigmoid function mentioned below.

7 $$\begin{aligned} \sigma = \frac{1}{1 + \text {e}^{-x}} \end{aligned}$$

The sigmoid function ensures that the output is constrained to the range [0,1], representing the probability of the sample being sensitive or resistant. The model is trained using the Adam optimizer [38] to reduce the binary cross-entropy loss function as follows:

8 $$\begin{aligned} \mathcal {L} = -\frac{1}{N}\sum _i^{N}(y_i\cdot \text {log}(\text {prob}(y_i)) + (1-y_i)\cdot \text {log}(1-\text {prob}(y_i))) \end{aligned}$$

where $y_i$ is the label of sample *i*, *N* is the total number of samples, and prob$(y_i)$ is the probability that sample *i* is sensitive or resistant as calculated by the sigmoid $\sigma$ activation function.

We have applied a pruning mechanism during the model construction to ensure biological consistency and efficiency. At first, genes without known protein interactions, proteins without associations to pathways, and pathways without drug targets are removed. Additionally, a backward pruning step is performed by iteratively discarding upstream nodes that become disconnected after the removal of downstream nodes. This process ensures that all retained nodes are part of a valid gene-drug path.

A primary advantage of BKDRP is enhanced interpretability compared to conventional deep learning models. A standard deep learning model has the capacity to provide feature importance at the input level only. Whereas BKDRP’s structured layers enable interpretation at four levels, i.e., input, protein, pathway, and drug. A detailed discussion of interpretability is presented in Sect. 3.6.

## Experimental results

This section describes the details of the experimental data used in this study and the results to validate our proposed method.

### Data preparation

This study utilizes the publicly available therapeutic genomic data from the GDSC database [7]. This database is chosen due to its extensive number of screened drugs. We have downloaded response measure data of anti-cancer drugs on in vitro cell lines from the GDSC (release 8.5, October 2023) database accessible at https://www.cancerrxgene.org/downloads/bulk_download. The molecular information of cell lines, such as RNA-seq gene expression profiles, mutation data, copy number variation, and protein data, have been downloaded from the same source, and the statistics of the dataset are given in Table 1. This table summarizes the data types, along with the number of cell lines and the number of genes or proteins.

**Table 1:** Description of different omics data

Data types	Cell lines	Genes/Proteins
RNA-seq gene expression data	1431	37602
Copy number variation	1398	777
Mutation	1399	612
Protein	948	8457

The downloaded drug response data covers 969 cell lines and 286 drugs with 242,036 log-normalized half-maximal inhibitory concentration (IC$_{50}$) values. We have meticulously chosen those cell lines that are annotated by the Cancer Genome Atlas (TCGA), which features a total of 30 different types of cancer from a total of 787 cell lines. Furthermore, we have collected 745 cell lines that are common across multiple datasets, including drug response data and all other omics data types. We have filtered 58 drugs that do not have a simplified molecular-input line-entry system (SMILES) from various sources such as DrugBank [39], PubChem [40], and ChEMBL [41]. The continuous values of IC$_{50}$ have been converted into binary values to make DRP a binary classification problem. Therefore, we binarize IC$_{50}$ values according to the paper mentioned in [15], where drug-resistant pairs are identified as having an IC$_{50}$ value higher than the maximum screening concentration that has been reported, and all other pairs are drug-sensitive pairs.

In this study, we obtain Transcripts Per Kilobase Million (TPM) values of gene expression data, and the values are log-transformed. In order to make expression profiles comparable for different platforms, the data are standardized, and a pairwise homogenization procedure is performed, as mentioned in [21, 42]. Moreover, we have excluded less informative genes, i.e., 50% of genes that exhibit lower variance [21]. Subsequently, we have selected the top 25% of these genes based on variance, resulting in a final set of 4680 genes. Using common cell lines, we have filtered the dataset to include 761 genes exhibiting copy number variation. Duplicate values corresponding to cell line and genes are aggregated. Further, we have removed genes with more than 5% of their values being 0, resulting in a final set of 716 genes. In a similar fashion, we have preprocessed the protein data. Initially, 8455 proteins are considered based on the common cell lines, and after all preprocessing tasks, we get 2422 proteins. For mutation data, VAF is considered, and we have taken 578 genes based on the common cell lines. Here, we have not performed any removal of mutated genes. In order to do a consistent comparison across all datasets, we have normalized each omics data individually using min-max scaling, ensuring their values range between 0 and 1.

### Experimental settings

The proposed method, BKDRP, is implemented using TensorFlow (version 2.12.0) with Keras (tf.keras) in Python version 3.10.18. The models are conducted on a 64-bit Windows workstation equipped with an AMD Ryzen 9 9900X 12-core processor (4.4 GHz), 32 GB RAM, and an NVIDIA RTX 5070 Ti GPU. Model development begins using preprocessed omics data comprising 4680 gene expression features, 716 CNV features, 578 mutation features, and 2422 protein features. In the first step, genes with no known associations to proteins are removed. Next, proteins unconnected to pathways are pruned. Likewise, pathways lacking connections to any drugs are also discarded, and only drugs that remain connected to at least one pathway are retained. Following this, a backward pruning strategy is applied: if the removal of downstream nodes (e.g., pathways/proteins) results in upstream nodes (e.g., proteins or genes) becoming disconnected, those upstream nodes are also iteratively removed. This ensures that all remaining nodes in the network are part of a connected path from gene to drug nodes. After pruning, the final feature vector for BKDRP consists of 837 features, comprising 442 gene expression, 211 CNV, 182 mutation, and 387 protein features. The layered structure of the BKDRP model includes 812 proteins in the first hidden layer, 51 pathways in the second layer, and 69 drugs in the last layer. Additionally, 1,024 drug features are used as input to the model. Table 2 summarizes dimensions of the mask and weight matrices, along with the number of input nodes and output nodes in each layer. The parameter settings of BKDRP are kept as batch size = 64, epochs = 50, and Adam optimizer with default learning rate = 0.001. For the details of the parameter setting experimentation of BKDRP, please see the supplementary file Table S1.

**Table 2:** Dimensions and connectivity of each layer in the proposed BKDRP

Layer connection	No. of input	No. of output	Matrix dimension ($M^{(l)}, W^{(l)}$)
Gene $\rightarrow$ Protein complex	837	812	837 $\times$ 812
Protein complex $\rightarrow$ Pathway	812	51	812 $\times$ 51
Pathway $\rightarrow$ Drug	51	69	$51 \times 69$
Drug $\rightarrow$ Output	69	1	$69 \times 1$

In-silico prediction of drug response to a novel experimental molecule can greatly benefit pharmaceutical research and drug design by reducing the high costs associated with large-scale drug screening. In order to evaluate the performance of the proposed method, we have designed a series of experiments. Similarly, predicting drug response for new cell lines can have clinical applications, facilitating oncologists in prioritizing treatment options for new patients by translating cell-line genomic data to patient genomic data. To address these scenarios, we have evaluated our method using four series of experimental designs: (i) predicting unknown drug–cell line responses, (ii) prediction of responses to new (unseen) drugs, (iii) prediction of responses to unseen cell lines, and (iv) performance analysis across multiple cancer types.

### Baseline methods

The following algorithms are used for comparative analysis. Model parameters are optimized through exhaustive experimentation using five-fold cross-validation, and the best-performing parameters are selected based on the AUC metric. The parameter settings results are given in the supplementary file Tables S2, S3, and S4.

- Ridge regression is a linear regression model with an L2 penalty. We have concatenated both the cell line features 1,222 (442 gene expression, 211 CNV, 182 mutation, and 387 protein features) and drug features (1024) and then have fed them into the model. It is implemented using the sklearn library [43]. We have tried different values of the L2 penalty coefficient from $\{0.1,0.5,1.0,5.0\}$ and selected the parameter where AUC is higher.
- Lasso regression, which employs L1 regularization, takes the same input and parameter settings as mentioned for ridge regression. Lasso regression is also implemented using the sklearn library [43].
- CNN baseline also takes the same input as mentioned earlier. We have performed a grid search over key hyperparameters, including convolutional filters (32, 64), (64, 128), kernel sizes 3, 5, 7, and learning rates 0.0001, 0.00001, 0.000001.
- tCNNS [18] model is applied to predict DRP using SMILES sequence of drugs and genomic mutation data of cancer cell lines. Here, the SMILES sequence will be encoded into a one-hot representation and fed into the neural network. We have kept the same hyperparameter settings as described in the original paper.

### Evaluation metrics

To evaluate the performance of the proposed model, we employ two evaluation metrics: AUC (Area Under the ROC Curve) and AUPR (Area Under the Precision-Recall Curve). AUC measures the area under the Receiver Operating Characteristic (ROC) curve, which plots the True Positive Rate (TPR) against the False Positive Rate (FPR) across different classification thresholds. The AUC value lies between 0 and 1, where a higher value indicates better performance of the model. On the other hand, AUPR represents the area under the precision-recall curve, highlighting the relationship between precision and recall. AUPR emphasizes the model’s performance in predicting positive instances, making it highly relevant to the goals of precision medicine. A higher AUPR value indicates better performance.

### Results

This section demonstrates the performance of BKDRP under different experimental settings. These experiments are designed to examine the model’s generalizability. The results are summarized as follows:

#### Predicting unknown drug–cell line responses

The first scenario of the experimental setting is used to train the model with known interactions of drug-cell line pairs and tested on the unknown pairs. Here, we have taken 31,560 resistant and 16,329 sensitive pairs from 30 different types of cancer and 69 drugs. The details of the cancer cell lines present are reported in the supplementary file Table S5. We have employed a five-fold cross-validation strategy by randomly splitting the drug-cell line pairs into five folds. Training is conducted in four folds, and the remaining one fold is used for testing. This process is repeated five times, with each fold serving as a test fold. This process guarantees that any drug-cell line pair can either exist in the training set or the testing set. However, there are no restrictions on the drug or cell lines. Fold-wise details of the result are given in supplementary Table S6.

**Table 3:** The performance comparison of AUC and AUPR for prediction of unknown drug–cell line combinations

Method	AUC	AUPR
Ridge	0.9152 ± 0.003	0.8595 ± 0.003
Lasso	0.9153 ± 0.003	0.8596 ± 0.003
CNN	0.6189 ± 0.093	0.4588 ± 0.1
tCNNS	0.9302 ± 0.003	0.8811 ± 0.006
BKDRP	0.9142 ± 0.004	0.8575 ± 0.007

This experiment aims to reflect the model’s capacity to generalize to unobserved drug–cell line interactions. Table 3 summarizes the AUC and AUPR of different methods along with BKDRP. The result clearly shows that the BKDRP (AUC: 0.9142 and AUPR: 0.8575) has achieved competitive performance comparable to classical regression methods such as Ridge and Lasso. This shows only marginal differences. An interesting performance hierarchy can be observed. While BKDRP does not become the top performer (tCNNS leads with 0.9302 AUC), it significantly outperforms the basic CNN approach. This suggests that BKDRP incorporates more sophisticated modeling techniques than standard convolutional networks.

#### Prediction of responses to new drugs

In the previous experiment, drug-cell line pairs have been randomly selected to be in the training set or the testing set. A drug or cell line may be present in both training and testing. This experimental setting can be beneficial in predicting a drug’s impact on a new cell line based on its observed response to a different one. The problem becomes more complicated when it is tested on a new drug/cell line, and its effect on any cell line/drug is not known. Therefore, in the second scenario, we focus on drug response prediction (DRP) for novel therapeutic compounds using an LODO strategy, which presents a critical challenge in precision oncology.

In each iteration, one drug is removed from the training dataset and used exclusively for testing, thereby evaluating the model’s ability to predict responses for previously unseen drugs. Hence, drug-cell line pairs are divided based on drugs. This partitioning ensures no information leakage regarding the target drug’s biological activity during model training. Table 4 shows the comparative performance across all methods for the LODO experiment. BKDRP achieves the highest AUC of 0.7880, demonstrating superior capacity for generalizing to unseen drugs compared to all baseline methods. The model marginally outperforms Lasso regression (AUC: 0.7878) while considerably surpassing deep learning approaches CNN and tCNNS by 30.1% and 25.6%, respectively. Although Lasso achieves slightly higher AUPR (0.5541 vs. 0.5529), the difference is negligible (0.2%), while BKDRP’s AUC advantage indicates better overall discriminative performance for novel drug prediction.

**Table 4:** The performance comparison of AUC and AUPR for the LODO experiment

Method	AUC	AUPR
Ridge	0.7770 ± 0.11	0.5486 ± 0.31
Lasso	0.7878 ± 0.11	0.5541 ± 0.31
CNN	0.6056 ± 0.13	0.4056 ± 0.31
tCNNS	0.6274 ± 0.14	0.4161 ± 0.30
BKDRP	0.7880 ± 0.10	0.5529 ± 0.31

Among the 69 drugs evaluated, BKDRP reveals heterogeneity in AUC scores ranging from 0.3666 to 0.9973. This can be found in the supplementary file Table S7. The highest predictive performance is observed for Motesanib, a multi-kinase inhibitor targeting FVEGR, RET, KIT, PDGFR, and RTK signaling pathway. Conversely, prediction challenges arise for Fulvestrant, which targets the estrogen receptor and acts through a hormone-related pathway. In addition to this, some high-performing drugs are Ribociclib, Avagacestat, Vismodegib, Bicalutamide, Palbociclib, Buparlisib, Bosutinib, Vinblastine, Dinaciclib. Drugs Ribociclib, Palbociclib, and Dinaciclib are inhibitors of cyclin-dependent kinases, highlighting the model’s strength in predicting responses to compounds targeting cell cycle regulation.

#### Prediction of responses to unseen cell lines

The third experimental scenario evaluates model performance in predicting drug responses for previously unseen cell lines using the LOCLO cross-validation strategy on the GDSC dataset. This evaluation setup reflects real-world precision medicine. In this setting, a single cell line is withheld during training and used for testing, generalizing the prediction for a novel cell line. Table 5 presents the comparative performance results for the LOCLO experiment. The results reveal high predictive performance across all methods, with AUC values exceeding 0.9 for traditional machine learning approaches. Ridge and Lasso regression show very similar performance (AUC 0.9096), while BKDRP shows competitive performance with an AUC of 0.9085, representing only a marginal 0.12% degradation from the top-performing methods.

**Table 5:** The performance comparison of AUC and AUPR for the LOCLO experiment

Method	AUC	AUPR
Ridge	0.9096 ± 0.05	0.8339 ± 0.12
Lasso	0.9096 ± 0.05	0.8337 ± 0.12
CNN	0.6825 ± 0.12	0.5614 ± 0.19
tCNNS	0.7058 ± 0.02	0.5190 ± 0.04
BKDRP	0.9085 ± 0.05	0.8338 ± 0.12

Table 6 illustrates the variability in BKDRP’s performance across the best and worst cell lines under the LOCLO experiment. The model achieves perfect predictive performance, i.e., AUC and AUPR as 1, for three different cell lines, including NCI-H2347 (LUAD: Lung Adenocarcinoma), AsPC-1 (PAAD: Pancreatic Adenocarcinoma), and BT-483 (BRCA: Breast Invasive Carcinoma). This indicates that BKDRP can generalize well to unseen cell lines that are biologically similar to those in the training set. However, in the case of ZR-75-30 (BRCA), the model exhibits noticeably lower performance (AUC = 0.642, AUPR = 0.289), highlighting the challenges of predicting drug response in certain cell lines. For more detailed results of each cell line, please see the supplementary file S8.

**Table 6:** Cell line-specific performance in LOCLO experimental settings

Cell Line ID	Cell line name	Cancer type	AUC	AUPR
SIDM00726	NCI-H2347	LUAD	1.000	1.000
SIDM00899	AsPC-1	PAAD	1.000	1.000
SIDM00892	BT-483	BRCA	1.000	1.000
SIDM00971	ZR-75-30	BRCA	0.642	0.289

#### Performance analysis across multiple cancer types

**Fig. 2:**
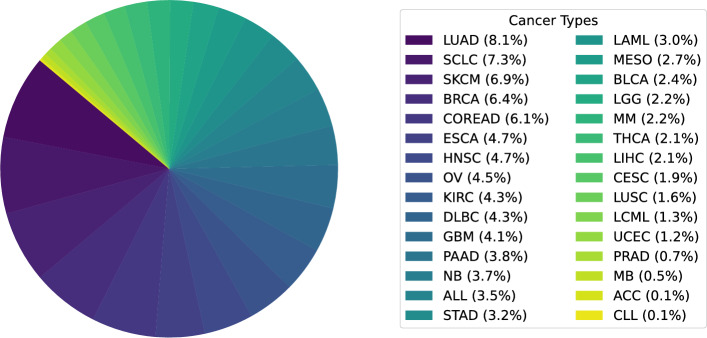
Distribution of cancer types in the GDSC dataset

The fourth experiment evaluates model generalization across distinct cancer types using the LOCO cross-validation strategy. In this scenario, all cell lines corresponding to a specific cancer type are excluded during training, and the model is subsequently evaluated on the withheld cancer types. The distribution of cancer types in the GDSC dataset is illustrated in Fig. 2. The pie chart shows the proportion of samples corresponding to each cancer type, with percentages indicated in the legend. Table 7 presents the overall performance comparison across all methods for the LOCO experiment, and details are given in the supplementary file Table S9. BKDRP consistently outperforms all baseline methods, achieving an AUC of 0.8845 and an AUPR of 0.8028. Specifically, BKDRP surpasses Ridge regression by 4.4% in AUC and 7.2% in AUPR, while significantly outperforming deep learning baselines CNN and tCNNS by 45.7% and 26.8% in AUC, respectively.

**Fig. 3:**
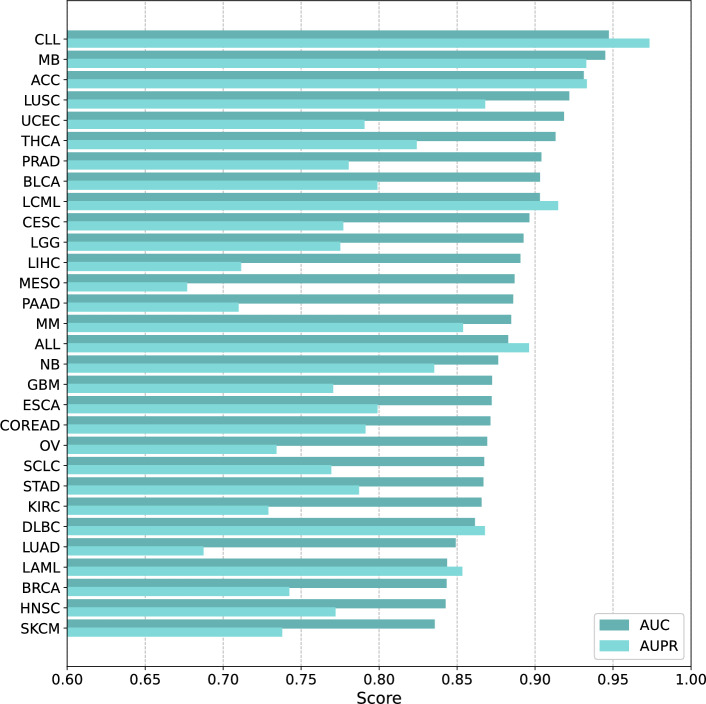
AUC and AUPR values of each cancer type in the LOCO setting

BKDRP achieves optimal prediction accuracy for Chronic Lymphocytic Leukemia (CLL) with an AUC of 0.9470, followed by Medulloblastoma (MB), Adrenocortical Carcinoma (ACC), and Lung Squamous Cell Carcinoma (LUSC). Conversely, the most challenging predictions occur for Skin Cutaneous Melanoma (SKCM) with an AUC of 0.8354, though this remains above 0.5. Figure 3 illustrates the performance distribution of each cancer type. The x-axis shows the corresponding AUC and AUPR scores, while the y-axis represents the different cancer types. Notably, even for the most challenging cancer type, SKCM, BKDRP maintains clinically relevant predictive performance (AUC > 0.8), demonstrating the robustness of the biologically informed architecture across diverse cancer contexts.

In addition to LOCLO, LODO, and LOCO, we have also conducted non-parametric alternatives, i.e., the Wilcoxon signed-rank test for the BKDRP with other baseline methods. The detailed results are provided in the supplementary file Table S10. From the results, it has been observed that BKDRP achieves a significant result compared to others.

**Table 7:** The performance comparison of AUC and AUPR for the LOCO experiment

Method	AUC	AUPR
Ridge	0.8475 ± 0.04	0.7487 ± 0.09
Lasso	0.8438 ± 0.05	0.7436 ± 0.08
CNN	0.6069 ± 0.10	0.4658 ± 0.15
tCNNS	0.6978 ± 0.03	0.5179 ± 0.11
BKDRP	0.8845 ± 0.03	0.8028 ± 0.08

### Biological interpretation: a case study of LUAD

To extract the biological insights of BKDRP, we have implemented a multi-level feature importance analysis on the trained model using the test dataset. Input gene importance is quantified using four methods: gradient-based analysis, activation-based scoring, variance analysis, and permutation. Here, *N* represents the number of test samples, $x_{ji}$ denotes the value of gene (feature) *i* in sample *j*, $L_j$ is the loss for sample *j*, $y_n$ is the true label of sample *n*, and $f(x_n)$ is the model prediction. The gradient-based feature importance is expressed as follows.

9 $$\begin{aligned} S_{\text {grad}}(i) = \frac{1}{N}\sum _{j=1}^N \left| \frac{\partial L(f(x_j),y_j)}{\partial x_{ji}} \right| \end{aligned}$$

where $\frac{\partial L_j}{\partial x_{ji}}$ quantifies the partial derivative of loss with respect to $x_{ji}$, indicating how much a small change in gene *i* value affects the model’s loss for sample *j* [44]. Equations (10) and (11) define the magnitude-based and variance-based analysis.

10 $$\begin{aligned} S_{\text {act}}(i) = \frac{1}{N}\sum _{j=1}^N |x_{ji}| \end{aligned}$$

11 $$\begin{aligned} \begin{aligned} S_{\text {var}}(i)&= \textrm{Var}(x_i) = \frac{1}{N-1}\sum _{j=1}^N (x_{ji} - \bar{x}_i)^2,\\ \bar{x}_i&= \frac{1}{N}\sum _j x_{ji} \end{aligned} \end{aligned}$$

Next, we use a permutation-based score that measures the change in the prediction error when the value of gene *i* is shuffled randomly. It is delineated in Eq. (12).

12 $$\begin{aligned} & \Delta _i = \text {error}_{\text {perm}}(i) - \text {error}_{\text {base}}, \\ & S_{\text {perm}}(i) = \max \big (0, \Delta _i\big ) \end{aligned}$$

Here the baseline error is defined as follows.

13 $$\begin{aligned} \text {error}_{\text {base}} = \frac{1}{N}\sum _{n=1}^N L(f(x_n),y_n) \end{aligned}$$

The overall combined score $S_{combined}$ is computed by integrating all four measures, as shown in Eq. (14).

14 $$\begin{aligned} S_{\text {combined}}(i) = \frac{1}{4} \left( \{S_m(i)\}_{m\in \text {grad}, \text {act}, \text {var}, \text {perm}}\right) \end{aligned}$$

To interpret the contributions of three layers in BKDRP, we have extracted the intermediate layer activations using TensorFlow’s functional API. In other words, we have created intermediate models that output activations from three biological layers: protein (812 nodes), pathway (51 nodes), and drug (69 nodes). For the individual layer, we have calculated activation-based scores to know the contribution of each biological entity in the final prediction. Let *l* be a layer with *p* nodes; the importance of node *k* is quantified across all *N* test samples using Eq. (15).

15 $$\begin{aligned} S_{layer}^l(k) = \frac{1}{N}\sum _{j=1}^N|a_{jk}^l| \end{aligned}$$

Where $a_{jk}^l$ denotes the activation of node *k* in *l* layer for *j* test sample. This gives us insights into the contribution of each protein, pathway, or drug that is activated while testing the sample. Hence, it is understood from the metric which biological component plays an important role in accurately predicting DRP. Based on the score, biological entities are ranked, and a higher value indicates greater importance in model prediction.

To analyze the biological interpretation, we have considered the most widely occurring cancer type as per the GDSC dataset 2, which is LUAD. In each of the 10 independent training runs, we have selected the top 20 biological entities based on their combined importance scores. These selections are then aggregated across all runs to identify the 10 most frequently occurring biological entities across genes, proteins, pathways, and drugs. The final top-ranked features, shown in Table 8, reflect consistent biological relevance in LUAD drug response. The most frequently identified genes are *KRAS*, *EGFR*, and *STK11*. These are well-known biomarkers and are mutated in Non-Small Cell Lung Cancer (NSCLC), as supported by previous studies [45–47]. *KRAS* mutations serve as a moderate but reliable indicator of poor clinical outcomes and limited treatment response in NSCLC. This creates the necessity for advancing targeted therapeutic strategies aimed at KRAS-mutant lung cancers [48].

Additionally, *SOCS1*, *HSPA8*, and *SMC3* are the most frequently occurring proteins. These proteins are implicated in cytokine signaling regulation, stress response, and chromosomal stability, respectively—processes critical to cancer progression [49]. Pathways cell cycle (hsa04110), PI3K-Akt signaling pathway (hsa04151), and pathways in cancer (hsa05200) are activated consistently across multiple runs. These pathways are widely reported to be dysregulated in LUAD and are common therapeutic targets. Ribociclib, Palbociclib, and Erlotinib have shown consistent importance in LUAD treatment. Among these drugs, Erlotinib is an EGFR tyrosine kinase inhibitor used to treat certain small cell lung cancers or advanced metastatic pancreatic cancers [39]. These findings not only validate the biological interpretability of BKDRP but also match well with known scientific and clinical knowledge, supporting its usefulness in personalized cancer treatment.

**Table 8:** Top 10 most influential biological entities identified through LOCO-based (LUAD) biological interpretation

Name	Top 10 entities
Gene	*KRAS*, *EGFR*, *STK11*, *DKC1*, *TRIM24*, *PSMD10*, *LRIG3*, *FGG*, *STAT3*, *CDK6*
Protein	*SOCS1*, *HSPA8*, *SMC3*, *CDKN2A*, *RFC5*, *SDC4*, *CTNNB1*, *PAX5*, *BMPR2*, *SQSTM1*
Pathway	hsa04110, hsa04151, hsa05200, hsa05202, hsa04510, hsa04380, hsa04150, hsa05225, hsa04915, hsa04014
Drug	Ribociclib, Sorafenib, Lenalidomide, Tretinoin, Vorinostat, Erlotinib, Foretinib, Avagacestat, Palbociclib, Afuresertib

### Ablation study

This section presents a systematic ablation study to evaluate the contribution of different combinations of omics data types to BKDRP’s predictive performance. We have conducted comprehensive experiments by systematically removing each omics data type while maintaining the biological architecture. In addition to this, we have also performed an ablation study to evaluate the performance of BKDRP with a similar capacity to a multi-layer perceptron (MLP). For this, we have used three hidden layers with the same number of nodes as used for BKDRP. We first took only cell line features, then considered cell line features concatenated with drug fingerprints to feed as input in the MLP. Table 9 summarizes the ablation results across five independent runs, reporting average performance metrics (accuracy, AUC, and AUPR) for each configuration. The experiments include using only gene expression data; removing copy number variation, mutations, and proteins one by one; and all four omics types (gene expression, mutations, copy number variations, and protein expression).

**Table 9:** Ablation study of BKDRP and Performance comparison with MLP

Omics data	Accuracy	AUC	AUPR
Only Gene Expression + BKDRP	0.8395 ± 0.0012	0.9117 ± 0.004	0.8535 ± 0.0009
w/o Copy Number Variation + BKDRP	0.8406 ± 0.0016	0.9134 ± 0.0002	0.8562 ± 0.0005
w/o Mutation + BKDRP	0.8412 ± 0.0011	0.9135 ± 0.0002	0.8565 ± 0.0004
w/o Protein + BKDRP	0.8416 ± 0.001	0.9133 ± 0.0002	0.8561 ± 0.0005
All omics + BKDRP	0.8417 ± 0.001	0.9137 ± 0.0004	0.8565 ± 0.0007
All omics + MLP	0.6674 ± 0.001	0.6525 ± 0.0009	0.4694 ± 0.0008
All omics + Fingerprints + MLP	0.8219 ± 0.0013	0.8445 ± 0.0047	0.8033 ± 0.0033

Several key aspects can be observed from the ablation study: removing any omics type results in consistent but modest decreases in all performance metrics, demonstrating that each omics contributes to the drug response accuracy. Notably, alone gene expression data provides a strong baseline performance, achieving 83.95% accuracy. However, integration of additional omics types enhances robustness, with the full multi-omics model achieving the best performance across all metrics. It can also be observed that BKDRP performs better than MLP. The detailed results for each run in every configuration are provided in the supplementary Table S11.

## Discussion

In this study, we have developed a model, termed BKDRP, a biologically informed neural network model for predicting anticancer drug response using multi-omics data and molecular fingerprints of drug molecules. BKDRP incorporates domain knowledge through a biologically meaningful graph structure, where nodes represent biological entities such as genes, proteins, pathways, and drugs; and edges represent the absence or presence of interactions. This model not only enhances prediction performance but also contributes to interpretability, which is a key aspect in precision oncology.

Our model demonstrates superior or comparable performance to existing methods across four different data split scenarios (Tables 3, 4, 5, and 7). Notably, BKDRP performs robustly on unseen cancer types, which shows that the model is capable of handling different and complex biological scenarios. The incorporation of biological information, such as protein-protein interactions and pathways, has proven crucial for improving robustness, especially in heterogeneous cell lines. Importantly, BKDRP offers biological interpretability, as shown in the results of the case study on LUAD. The results highlight key nodes (e.g., genes, proteins, pathways, and drugs) that drive drug response. These biomarker candidates have been validated against existing literature.

Though BKDRP performs well, there remains room for enhancement. In some instances, its performance is on par with baseline methods. Future work may focus on integrating additional drug-specific features—such as detailed structural information of drugs and drug-target interactions. Expanding the training dataset to include a wider chemical and biological diversity of drugs and cell lines could further improve model generalizability. In conclusion, BKDRP represents a promising step toward interpreting and accurately predicting drug responses.

## Conclusion

The proposed method BKDRP, a biologically knowledge-driven drug response prediction model, integrates multi-omics data and drug molecular fingerprints within a biologically constrained deep neural network architecture. BKDRP is modelled as gene-protein-pathway-drug hierarchy, which achieves not only competitive accuracy across different evaluation strategies but also offers biological interpretability. The case study reveals the ability of BKDRP to identify relevant biomarkers, proteins, pathways, and drugs. It can be helpful in personalized cancer therapy, drug prioritization, and biomarker discovery.

## Additional file

Supplementary file 1 (pdf 201 KB)

## Data Availability

The GDSC dataset, including gene expression data, protein expression, copy number variation, and mutation data used in this study, can be downloaded from the link https://www.cancerrxgene.org/downloads/bulk_download. The code and the preprocessed data are available at https://github.com/Koyel-Mandal/BKDRP.git.
