# Supplementary material for: BKDRP: a biological knowledge-driven approach for drug response prediction using multi-omics data in cancer cell lines
Source: BMC Bioinformatics. 2026 Mar 17;27:119. doi: 10.1186/s12859-026-06406-2 (PMC13235134; doi:10.1186/s12859-026-06406-2)
Supplement: Supplementary file 1 — (pdf 201 KB) [file 12859_2026_6406_MOESM1_ESM.pdf]

Supplementary

BKDRP: a biological knowledge-driven approach for drug response prediction using multi-omics data in cancer cell lines

Koyel Mandal[1] and Sanghamitra Bandyopadhyay[1,*]

[1]Indian Statistical Institute, Machine Intelligence Unit, Kolkata, 700108, India
[*]sanghami@isical.ac.in

Parameter settings for different methods: The parameter settings of the different methods, BKDRP, Ridge Regression, Lasso Regression, and CNN are summarized in Table S1, S2, S3, and S4, respectively. Model parameters are optimized through exhaustive experimentation using a single five-fold cross-validation, and the best-performing parameters are selected based on the AUC metric. Moreover, we have used the same random seed while conducting the five-fold cross-validation split. The selected parameters and their corresponding values are highlighted in bold.

Table S1: Performance of BKDRP with varying batch size using 5-fold cross-validation.

Batch size	AUC	AUPR
32	0.9127	0.8556
**64**	**0.9142**	**0.8575**
128	0.9140	0.8572

Note: Bold values indicate the best performance across different batch sizes.

Table S2: Performance of Ridge Regression with varying coefficient using 5-fold cross-validation

Coefficient	AUC	AUPR
**0.1**	**0.9152**	**0.8595**
0.5	0.9151	0.8593
1	0.9151	0.8593
5	0.9151	0.8593

Note: Bold values indicate the best performance across different coefficients.

Table S3: Performance of Lasso Regression with varying coefficient using 5-fold cross-validation

Coefficient	AUC	AUPR
0.1	0.915	0.859
**0.5**	**0.9153**	**0.8596**
1	0.9152	0.8595
5	0.9151	0.8594

Note: Bold values indicate the best performance across different coefficients.

Summary of cancer types: The details of the cancer types are shown in Table S5.

Experimental results: The detailed results of five-fold cross-validation for predicting unknown drug-cell line pairs, LODO, LOCLO, and LOCO are summarized in Table S6, S7, S8, and S9, respectively.

Table S4: Performance of Convolutional Neural Network with varying different parameters using 5-fold cross-validation

Filter1	Filter2	Kernel	Learning rate	AUC	AUPR
**32**	**64**	**3**	**1.00E-03**	**0.6392**	**0.4682**
32	64	3	1.00E-04	0.5835	0.4069
32	64	3	1.00E-05	0.4536	0.3112
32	64	5	1.00E-03	0.6245	0.467
32	64	5	1.00E-04	0.562	0.3928
32	64	5	1.00E-05	0.4523	0.3106
32	64	7	1.00E-03	0.5706	0.4029
32	64	7	1.00E-04	0.5927	0.417
32	64	7	1.00E-05	0.4492	0.3091
64	128	3	1.00E-03	0.5976	0.4352
64	128	3	1.00E-04	0.5746	0.3971
64	128	3	1.00E-05	0.4541	0.3111
64	128	5	1.00E-03	0.5269	0.3689
64	128	5	1.00E-04	0.5544	0.39
64	128	5	1.00E-05	0.4732	0.327
64	128	7	1.00E-03	0.5328	0.3672
64	128	7	1.00E-04	0.5887	0.421
64	128	7	1.00E-05	0.4675	0.3215

Note: Bold values indicate the best performance across different parameter combinations.

Table S5: Summary of cancer types, including number of cell lines, drugs, and drug–cell line pairs for each cancer type.

Abbreviation	Description	No. of cell line	No. of Drugs	Drug-cell line
LUAD	Lung adenocarcinoma	60	69	3869
SCLC	Small Cell Lung Cancer	56	69	3503
SKCM	Skin Cutaneous Melanoma	51	69	3282
BRCA	Breast invasive carcinoma	47	69	3071
COREAD	Colon and Rectal Adenocarcinoma	45	69	2944
ESCA	Esophageal carcinoma	35	69	2257
HNSC	Head and Neck squamous cell carcinoma	35	69	2255
OV	Ovarian serous cystadenocarcinoma	34	69	2164
KIRC	Kidney renal clear cell carcinoma	32	69	2066
DLBC	Lymphoid Neoplasm Diffuse Large B-cell Lymphoma	32	69	2046
GBM	Glioblastoma multiforme	31	69	1985
PAAD	Pancreatic adenocarcinoma	28	69	1799
NB	Neuroblastoma	28	69	1774
ALL	Acute lymphoblastic leukemia	26	69	1669
STAD	Stomach adenocarcinoma	24	69	1524
LAML	Acute Myeloid Leukemia	23	69	1449
MESO	Mesothelioma	20	69	1310
BLCA	Bladder Urothelial Carcinoma	18	69	1165
LGG	Brain Lower Grade Glioma	17	69	1064
MM	Multiple Myeloma	16	69	1048
THCA	Thyroid carcinoma	16	69	1027
LIHC	Liver hepatocellular carcinoma	15	69	990
CESC	Cervical and endocervical cancers	14	69	921
LUSC	Lung squamous cell carcinoma	12	66	790
LCML	Chronic Myelogenous Leukemia	10	66	613
UCEC	Uterine Corpus Endometrial Carcinoma	9	66	593
PRAD	Prostate adenocarcinoma	5	66	330
MB	Medulloblastoma	4	66	251
ACC	Adrenocortical carcinoma	1	66	66
CLL	Chronic Lymphocytic Leukemia	1	64	64

Table S6: Drug-cell line performance (AUC and AUPR) of BKDRP and baseline methods across five-fold cross-validation.

Fold	Ridge		Lasso		CNN		tCNNS		BKDRP	
	AUC	AUPR	AUC	AUPR	AUC	AUPR	AUC	AUPR	AUC	AUPR
1	0.9157	0.8617	0.9158	0.8618	0.6809	0.5187	0.9358	0.8904	0.919922	0.867834
2	0.9203	0.8629	0.9204	0.8631	0.734	0.5964	0.9273	0.8749	0.910528	0.849021
3	0.9137	0.8585	0.9137	0.8585	0.4922	0.3395	0.9287	0.8782	0.91537	0.857331
4	0.9148	0.8579	0.9149	0.858	0.5962	0.4249	0.9293	0.8797	0.912941	0.856785
5	0.9116	0.8563	0.9116	0.8564	0.5911	0.4146	0.9278	0.8822	0.912037	0.856448

Table S7: LODO performance (AUC and AUPR) of BKDRP and baseline methods across drugs.

Drug	Ridge		Lasso		CNN		tCNN		BKDRP	
	AUC	AUPR	AUC	AUPR	AUC	AUPR	AUC	AUPR	AUC	AUPR
Afatinib	0.7832	0.8593	0.7824	0.86	0.4402	0.6231	0.5907	0.7056	0.7799	0.8587
Afuresertib	0.779	0.7236	0.7796	0.7244	0.6251	0.61	0.6463	0.557	0.7786	0.7178
Alpelisib	0.7816	0.3745	0.7789	0.3677	0.4386	0.1144	0.6113	0.1981	0.7802	0.3669
Avagacestat	0.9549	0.1885	0.9512	0.1702	0.9393	0.0872	0.6647	0.0085	0.942	0.1846
Axitinib	0.8234	0.687	0.8262	0.696	0.7475	0.614	0.7523	0.4955	0.8196	0.6873
Bicalutamide	0.9357	0.0833	0.924	0.0714	0.9181	0.0667	0.9327	0.0417	0.924	0.0714
Bortezomib	0.8057	0.9458	0.8062	0.9466	0.6117	0.8708	0.7188	0.7188	0.8005	0.9451
Bosutinib	0.899	0.5352	0.9016	0.542	0.7056	0.1846	0.7937	0.2138	0.9026	0.5363
Buparlisib	0.9167	0.987	0.9173	0.9872	0.603	0.9135	0.232	0.8945	0.915	0.9867
Cediranib	0.8051	0.8464	0.8039	0.8461	0.6673	0.7477	0.6268	0.6817	0.7988	0.8401
Crizotinib	0.8636	0.814	0.8673	0.8206	0.6986	0.5841	0.6758	0.5844	0.8702	0.8277
Dabrafenib	0.7054	0.3314	0.7036	0.3263	0.6187	0.2243	0.6598	0.6598	0.7021	0.3252
Dactolisib	0.8192	0.8309	0.8171	0.8279	0.5304	0.5661	0.6408	0.6247	0.8161	0.8245
Dasatinib	0.5916	0.4409	0.5869	0.4358	0.4588	0.335	0.5608	0.3992	0.5756	0.4276
Dinaciclib	0.8857	0.9886	0.8888	0.989	0.5962	0.9485	0.5786	0.9431	0.8869	0.9889
Docetaxel	0.873	0.8763	0.8776	0.8802	0.6051	0.5869	0.6112	0.6112	0.8742	0.8782
Doramapimod	0.7982	0.1453	0.7914	0.136	0.694	0.0662	0.6015	0.0559	0.7923	0.1375
Entinostat	0.7878	0.8243	0.788	0.8254	0.4528	0.509	0.6916	0.6746	0.7848	0.8194
Entospletinib	0.8132	0.369	0.8106	0.3606	0.6431	0.1713	0.6501	0.1896	0.8098	0.3578
Erlotinib	0.715	0.2337	0.711	0.2265	0.5513	0.1747	0.5563	0.1346	0.7155	0.2403
Fludarabine	0.7462	0.362	0.7455	0.3645	0.3789	0.1163	0.7197	0.3193	0.7595	0.3752
Foretinib	0.8625	0.9757	0.863	0.9756	0.6555	0.9195	0.5978	0.894	0.8599	0.9751
Fulvestrant	0.3369	0.002	0.3302	0.002	0.7022	0.0045	0.4104	0.0013	0.3666	0.0021
Gefitinib	0.6817	0.0806	0.6788	0.0796	0.4891	0.049	0.6302	0.0755	0.6868	0.081
Gemcitabine	0.7689	0.9263	0.7668	0.9253	0.542	0.8208	0.651	0.8554	0.762	0.9234
Ibrutinib	0.7492	0.3909	0.7469	0.3886	0.6469	0.3046	0.5439	0.2025	0.7565	0.388
Ipatasertib	0.7629	0.4495	0.7614	0.4484	0.6372	0.3357	0.6475	0.2907	0.7547	0.4415
Lapatinib	0.742	0.513	0.7407	0.5116	0.6043	0.3995	0.5639	0.3403	0.7492	0.5239
Leflunomide	0.7735	0.6357	0.7743	0.6402	0.6312	0.3605	0.6869	0.4569	0.7869	0.6589
Lenalidomide	0.8425	0.0207	0.8425	0.0215	0.7241	0.0119	0.8156	0.0145	0.8415	0.0207
Lestaurtinib	0.8566	0.9161	0.8569	0.9169	0.6646	0.7794	0.5387	0.6758	0.8597	0.9187
Linsitinib	0.7526	0.2349	0.7538	0.2319	0.6102	0.146	0.5694	0.1267	0.7406	0.2276
Luminespib	0.8406	0.9551	0.8384	0.9542	0.5328	0.8401	0.5672	0.8429	0.8366	0.9538
Methotrexate	0.5818	0.4598	0.5807	0.4615	0.5161	0.4364	0.531	0.4135	0.5861	0.474
Motesanib	0.9964	0.5917	0.9964	0.5873	0.9475	0.0959	0.8451	0.013	0.9973	0.6333
Mycophenolic acid	0.8091	0.8586	0.8107	0.8593	0.665	0.7478	0.5191	0.6306	0.8093	0.8585
Nilotinib	0.7613	0.3275	0.7576	0.3235	0.4643	0.1135	0.5503	0.1349	0.7601	0.3218
Niraparib	0.8285	0.3943	0.8259	0.3878	0.6771	0.1739	0.8168	0.3215	0.8202	0.3697
Obatoclax Mesylate	0.8617	0.9873	0.8637	0.9877	0.5613	0.9366	0.6639	0.9511	0.8616	0.9875
Olaparib	0.8459	0.291	0.8439	0.2932	0.7581	0.1684	0.6664	0.1143	0.8466	0.2919
Osimertinib	0.6641	0.1656	0.6617	0.1621	0.559	0.1468	0.531	0.117	0.6574	0.1569
Paclitaxel	0.7603	0.6676	0.7606	0.6693	0.5558	0.413	0.678	0.5077	0.7517	0.6499

Continued on next page

Table S7 – continued from previous page

Drug	Ridge		Lasso		CNN		tCNN		BKDRP	
	AUC	AUPR	AUC	AUPR	AUC	AUPR	AUC	AUPR	AUC	AUPR
Palbociclib	0.9198	0.7095	0.9194	0.7158	0.7039	0.3668	0.3691	0.2257	0.915	0.7072
Pictilisib	0.7692	0.8986	0.7663	0.8974	0.6261	0.8235	0.5169	0.7614	0.7635	0.896
Rapamycin	0.7647	0.3661	0.7633	0.3618	0.587	0.2117	0.6441	0.2543	0.7588	0.358
Ribociclib	0.973	0.5533	0.9757	0.4648	0.7215	0.0185	0.7997	0.0167	0.9749	0.3973
Romidepsin	0.7827	0.9139	0.7827	0.913	0.6479	0.8335	0.596	0.8066	0.7822	0.9128
Rucaparib	0.846	0.1839	0.8424	0.1736	0.2666	0.0096	0.6732	0.0453	0.8639	0.2138
Ruxolitinib	0.8027	0.2088	0.8068	0.212	0.7893	0.0653	0.6345	0.0183	0.8045	0.2225
Savolitinib	0.5648	0.0123	0.5595	0.0119	0.526	0.0116	0.4849	0.0095	0.5379	0.0111
Selumetinib	0.6529	0.407	0.6503	0.4018	0.5012	0.2529	0.5825	0.301	0.6459	0.3984
Sepantronium bromide	0.6689	0.9982	0.6771	0.9983	0.4319	0.996	0.0529	0.9961	0.6866	0.9984
Sorafenib	0.7909	0.7202	0.7927	0.7273	0.6353	0.5296	0.709	0.5545	0.7917	0.7245
Talazoparib	0.799	0.6425	0.7973	0.6398	0.4339	0.2876	0.5776	0.3964	0.7961	0.6391
Tamoxifen	0.8347	0.2576	0.8359	0.2631	0.6506	0.1273	0.7425	0.1753	0.8406	0.2621
Tanespimycin	0.7278	0.7312	0.7259	0.7284	0.5438	0.5911	0.6291	0.6381	0.7133	0.7112
Taselisib	0.7782	0.7906	0.7759	0.7908	0.4059	0.6713	0.6624	0.6995	0.7757	0.7852
Temsirolimus	0.7672	0.4066	0.7623	0.4046	0.5732	0.284	0.7063	0.2798	0.7618	0.3912
Tozasertib	0.8233	0.7481	0.8217	0.7447	0.4434	0.3667	0.7163	0.5448	0.8372	0.7529
Trametinib	0.6077	0.5239	0.6045	0.5193	0.497	0.4266	0.5289	0.443	0.6045	0.5159
Tretinoin	0.789	0.1412	0.787	0.0976	0.7692	0.0597	0.8639	0.0612	0.8008	0.1154
Ulixertinib	0.734	0.5839	0.7323	0.5797	0.541	0.3754	0.6458	0.4466	0.7272	0.5719
Venetoclax	0.7331	0.2773	0.7346	0.281	0.7408	0.2097	0.5248	0.1023	0.7396	0.2734
Vinblastine	0.8939	0.9492	0.8928	0.949	0.6698	0.8042	0.6023	0.7423	0.8888	0.9464
Vinorelbine	0.8789	0.9306	0.8812	0.9328	0.5089	0.6596	0.8344	0.8604	0.8801	0.9316
Vismodegib	0.9105	0.11	0.9252	0.1381	0.6364	0.0271	0.8367	0.0164	0.9293	0.1055
Vorinostat	0.8564	0.9494	0.8563	0.9493	0.6575	0.8491	0.5616	0.7894	0.8575	0.9494

Table S8: LOCLO performance (AUC and AUPR) of BKDRP and baseline methods across cancer cell line.

Cell line	Ridge		Lasso		CNN		tCNN		BKDRP	
	AUC	AUPR	AUC	AUPR	AUC	AUPR	AUC	AUPR	AUC	AUPR
SIDM01132	0.904	0.9091	0.904	0.9091	0.5693	0.6095	0.7593	0.7052	0.9067	0.9075
SIDM00909	0.8747	0.6939	0.8747	0.6939	0.6773	0.3756	0.72	0.41	0.8693	0.7086
SIDM01085	0.8783	0.7684	0.8783	0.7684	0.7065	0.565	0.712	0.4508	0.8674	0.7541
SIDM01160	0.9437	0.8268	0.9437	0.8268	0.8365	0.6579	0.647	0.319	0.9437	0.8267
SIDM01190	0.9686	0.9177	0.9686	0.9177	0.7961	0.6402	0.7725	0.4295	0.9686	0.9177
SIDM00889	0.9093	0.9053	0.9093	0.9053	0.8343	0.8563	0.6694	0.624	0.9009	0.8972
SIDM00905	0.9841	0.9697	0.9841	0.9697	0.9238	0.9207	0.7698	0.5446	0.9767	0.9548
SIDM00627	0.8999	0.8703	0.8999	0.8703	0.7958	0.7402	0.7544	0.6407	0.9029	0.874
SIDM00998	0.937	0.9393	0.937	0.9393	0.6889	0.6712	0.7763	0.6604	0.937	0.9407
SIDM00799	0.9573	0.9443	0.9573	0.9443	0.6828	0.6824	0.6599	0.4516	0.9554	0.9443
SIDM00581	0.945	0.8689	0.945	0.8689	0.8225	0.6717	0.7053	0.5222	0.94	0.8543
SIDM01171	0.9642	0.9149	0.9642	0.9149	0.7962	0.7157	0.7833	0.5187	0.9698	0.9292
SIDM01193	0.9217	0.8789	0.9217	0.8789	0.8022	0.7057	0.7911	0.5659	0.9185	0.8753
SIDM00922	0.8187	0.6673	0.8187	0.6673	0.5834	0.3673	0.6717	0.3981	0.8055	0.6467
SIDM00548	0.9474	0.892	0.9474	0.892	0.8686	0.8276	0.6815	0.4158	0.9403	0.8797
SIDM00747	0.8298	0.7104	0.8287	0.7083	0.6137	0.4816	0.6596	0.3969	0.832	0.7149
SIDM00746	0.8323	0.8482	0.8323	0.8482	0.5432	0.5818	0.7005	0.4399	0.8335	0.8461
SIDM00745	0.9167	0.7913	0.9167	0.7913	0.6929	0.4474	0.6783	0.4182	0.9167	0.7849
SIDM00742	0.9041	0.6287	0.9041	0.6287	0.881	0.5866	0.6871	0.4198	0.9091	0.6672
SIDM00769	0.8805	0.7121	0.8805	0.7121	0.7044	0.397	0.6799	0.4169	0.8679	0.7083
SIDM00709	0.9095	0.6133	0.9095	0.6133	0.6315	0.2249	0.6927	0.432	0.9159	0.6173
SIDM00716	0.9118	0.92	0.9118	0.92	0.6129	0.5672	0.6909	0.4156	0.908	0.9154
SIDM00729	0.9111	0.7235	0.9111	0.7235	0.8092	0.6156	0.6914	0.4198	0.9098	0.7479

Continued on next page

Cell line	Ridge		Lasso		CNN		tCNN		BKDRP	
	AUC	AUPR	AUC	AUPR	AUC	AUPR	AUC	AUPR	AUC	AUPR
SIDM00727	0.9033	0.6516	0.9033	0.6516	0.7333	0.3761	0.7257	0.508	0.8967	0.6751
SIDM00726	1	1	1	1	0.8889	0.5714	0.7026	0.4851	1	1
SIDM00724	0.8197	0.3988	0.8197	0.3988	0.8131	0.2254	0.6981	0.4839	0.8197	0.4165
SIDM00509	0.9276	0.9301	0.9276	0.9301	0.86	0.8558	0.7125	0.4882	0.9258	0.9317
SIDM00865	0.985	0.964	0.985	0.964	0.8912	0.8166	0.6861	0.4729	0.9815	0.9514
SIDM00522	0.9585	0.9623	0.9585	0.9623	0.8175	0.8017	0.7051	0.5008	0.9558	0.9584
SIDM00654	0.9039	0.8476	0.9039	0.8476	0.6754	0.5628	0.7203	0.5161	0.9029	0.8517
SIDM00653	0.9173	0.8464	0.9173	0.8464	0.6197	0.4556	0.6984	0.4905	0.9202	0.8351
SIDM00741	0.9222	0.8232	0.9222	0.8232	0.5639	0.3719	0.7024	0.4863	0.9208	0.807
SIDM00760	0.9286	0.8913	0.9286	0.8913	0.5881	0.5119	0.6778	0.4801	0.9286	0.8907
SIDM00706	0.8525	0.8779	0.8525	0.8779	0.6194	0.6206	0.7204	0.5067	0.8525	0.8799
SIDM00699	0.9564	0.7167	0.9564	0.7167	0.7748	0.3564	0.7125	0.4986	0.9613	0.7269
SIDM00965	0.9636	0.9583	0.9636	0.9583	0.8994	0.889	0.6865	0.471	0.9598	0.9548
SIDM01128	0.897	0.8718	0.897	0.8718	0.513	0.4973	0.7178	0.5037	0.893	0.8581
SIDM01126	0.8625	0.6805	0.8625	0.6805	0.7984	0.5046	0.6985	0.4755	0.8531	0.6523
SIDM01121	0.9492	0.9441	0.9492	0.9441	0.6589	0.5395	0.7032	0.4898	0.9492	0.9445
SIDM01131	0.9356	0.9214	0.9356	0.9214	0.6644	0.5812	0.7038	0.4908	0.9346	0.9184
SIDM00713	0.9485	0.8225	0.9485	0.8225	0.7671	0.6005	0.7207	0.5181	0.9451	0.8161
SIDM01100	0.9208	0.9228	0.9208	0.9228	0.6701	0.6132	0.7264	0.5048	0.9171	0.9202
SIDM01099	0.899	0.7733	0.899	0.7733	0.5349	0.3559	0.7125	0.4986	0.8976	0.7669
SIDM00320	0.9231	0.7631	0.9231	0.7631	0.6792	0.4667	0.6474	0.457	0.91	0.742
SIDM00368	0.9586	0.9413	0.9586	0.9413	0.7246	0.6893	0.647	0.4677	0.9539	0.9349
SIDM00294	0.9752	0.96	0.9752	0.96	0.4653	0.385	0.6821	0.4931	0.9712	0.9525
SIDM01098	0.9444	0.9103	0.9444	0.9103	0.8944	0.8344	0.6276	0.47	0.9472	0.9138
SIDM00702	0.9624	0.974	0.9624	0.974	0.766	0.8457	0.6082	0.4609	0.9633	0.975
SIDM01124	0.9725	0.8921	0.9725	0.8921	0.8462	0.7146	0.6655	0.5178	0.9657	0.8752
SIDM01148	0.9756	0.9833	0.9756	0.9833	0.6472	0.6948	0.681	0.4196	0.9542	0.9709
SIDM01101	0.963	0.9412	0.963	0.9412	0.9122	0.8802	0.6893	0.4259	0.9672	0.9469
SIDM00708	0.9303	0.8115	0.9303	0.8115	0.8824	0.6897	0.6801	0.419	0.9216	0.8053
SIDM01182	0.9252	0.7533	0.9252	0.7533	0.5867	0.4259	0.6976	0.443	0.9235	0.7507
SIDM01174	0.9683	0.6806	0.9683	0.6806	0.7354	0.1315	0.6918	0.4315	0.9683	0.6806
SIDM00921	0.9174	0.8161	0.9174	0.8161	0.7652	0.6615	0.6732	0.4085	0.9141	0.8123
SIDM00645	0.9075	0.7656	0.9075	0.7656	0.7475	0.6796	0.6768	0.4216	0.9088	0.7734
SIDM00771	0.9502	0.9212	0.9502	0.9212	0.6848	0.7369	0.6783	0.4307	0.953	0.9282
SIDM00505	0.946	0.8597	0.946	0.8597	0.7551	0.6058	0.6962	0.4332	0.9424	0.8496
SIDM01078	0.9632	0.925	0.9632	0.925	0.5714	0.4274	0.6886	0.4264	0.9567	0.9068
SIDM00756	0.9465	0.9214	0.9465	0.9214	0.6096	0.5602	0.7007	0.4457	0.9479	0.9245
SIDM01163	0.9148	0.6233	0.9148	0.6233	0.7705	0.2039	0.6767	0.4231	0.9049	0.6093
SIDM00347	0.9281	0.7671	0.9281	0.7671	0.8471	0.632	0.6953	0.4248	0.9216	0.7463
SIDM00872	0.8717	0.7948	0.8717	0.7948	0.6404	0.4621	0.7156	0.4488	0.8717	0.7985
SIDM00866	0.9138	0.8223	0.9138	0.8223	0.6236	0.3484	0.6778	0.4169	0.9036	0.8077
SIDM00885	0.8846	0.7313	0.8846	0.7313	0.5583	0.2662	0.6852	0.4264	0.8833	0.7202
SIDM00884	0.8889	0.806	0.8889	0.806	0.8208	0.6205	0.7171	0.4527	0.8958	0.8143
SIDM00874	0.9188	0.68	0.9188	0.68	0.7565	0.3519	0.6945	0.432	0.9156	0.6815
SIDM00774	0.9014	0.8655	0.9014	0.8655	0.6874	0.5751	0.6993	0.4442	0.9024	0.8623
SIDM00675	0.9212	0.7222	0.9212	0.7222	0.6439	0.3199	0.6846	0.4231	0.9076	0.728
SIDM00489	0.891	0.8883	0.891	0.8883	0.7055	0.7462	0.7856	0.6448	0.8966	0.8922
SIDM00942	0.9073	0.8686	0.9073	0.8686	0.7122	0.5679	0.6952	0.579	0.9044	0.866
SIDM00953	0.9539	0.9472	0.9539	0.9472	0.5367	0.4594	0.7046	0.6464	0.9558	0.9507
SIDM00951	0.8977	0.9134	0.8977	0.9134	0.5889	0.6416	0.6822	0.6201	0.9041	0.9177
SIDM00970	0.9058	0.8944	0.9058	0.8944	0.776	0.6821	0.7148	0.5357	0.8913	0.8736
SIDM00486	0.918	0.8929	0.918	0.8929	0.7971	0.7221	0.7179	0.5335	0.9132	0.8919
SIDM00278	0.9269	0.9469	0.9269	0.9469	0.713	0.7766	0.7089	0.5369	0.9269	0.9457
SIDM01044	0.9385	0.904	0.9385	0.904	0.8577	0.8199	0.6989	0.5239	0.9394	0.91
SIDM00591	0.8877	0.9314	0.8877	0.9314	0.5174	0.6517	0.7092	0.5315	0.885	0.9312

Continued on next page

Cell line	Ridge		Lasso		CNN		tCNN		BKDRP	
	AUC	AUPR	AUC	AUPR	AUC	AUPR	AUC	AUPR	AUC	AUPR
SIDM00590	0.9361	0.924	0.9361	0.924	0.8361	0.8504	0.7002	0.5295	0.9306	0.9208
SIDM00632	0.9364	0.9539	0.9364	0.9539	0.7968	0.8854	0.7073	0.5337	0.943	0.9593
SIDM00230	0.8866	0.889	0.8866	0.889	0.7938	0.7822	0.706	0.523	0.883	0.887
SIDM00693	0.8752	0.5696	0.8752	0.5696	0.6589	0.3582	0.6964	0.5377	0.885	0.6327
SIDM00606	0.9205	0.824	0.9205	0.824	0.8723	0.7862	0.7051	0.5373	0.9127	0.8091
SIDM00301	0.9444	0.8996	0.9444	0.8996	0.8028	0.777	0.6789	0.5061	0.9403	0.8974
SIDM01032	0.8807	0.8745	0.8807	0.8745	0.6095	0.5953	0.6858	0.5113	0.8863	0.8781
SIDM01028	0.89	0.7647	0.89	0.7647	0.7407	0.5483	0.7102	0.5246	0.8843	0.7532
SIDM01025	0.8241	0.656	0.8229	0.6375	0.6586	0.4075	0.676	0.503	0.8241	0.6538
SIDM01024	0.9536	0.8311	0.9536	0.8311	0.9161	0.7814	0.696	0.5208	0.9446	0.8124
SIDM00182	0.9089	0.8218	0.9089	0.8218	0.7839	0.5728	0.667	0.4941	0.9143	0.816
SIDM00181	0.9676	0.928	0.9676	0.928	0.8148	0.7642	0.6974	0.5248	0.9688	0.9322
SIDM00180	0.9126	0.8966	0.9126	0.8966	0.7284	0.7083	0.6871	0.5096	0.9173	0.9007
SIDM00292	0.9274	0.4958	0.9274	0.4958	0.8105	0.2492	0.7269	0.5473	0.9274	0.4958
SIDM00339	0.9779	0.9686	0.9779	0.9686	0.8192	0.7653	0.6889	0.5191	0.9817	0.9727
SIDM00245	0.9102	0.8552	0.9102	0.8552	0.6254	0.5218	0.7006	0.5275	0.9083	0.8502
SIDM00751	0.8873	0.6953	0.8873	0.6953	0.6358	0.2993	0.7106	0.5408	0.8873	0.6941
SIDM00237	0.9017	0.9172	0.9017	0.9172	0.3555	0.4544	0.6908	0.5225	0.9034	0.9172
SIDM00367	0.9229	0.9327	0.9229	0.9327	0.5733	0.5456	0.7048	0.5259	0.9219	0.9318
SIDM00391	0.8407	0.7153	0.8407	0.7153	0.8695	0.7468	0.6902	0.5223	0.8516	0.7296
SIDM00417	0.8431	0.5721	0.8431	0.5721	0.3804	0.2507	0.6979	0.5184	0.8536	0.6029
SIDM01229	0.9529	0.9507	0.9529	0.9507	0.5817	0.5461	0.6874	0.5098	0.9538	0.9446
SIDM01009	0.9417	0.9347	0.9417	0.9347	0.7083	0.6729	0.6959	0.5227	0.9444	0.9375
SIDM00395	0.9335	0.9136	0.9335	0.9136	0.631	0.6167	0.6935	0.5208	0.9315	0.9151
SIDM00369	0.908	0.8592	0.908	0.8592	0.6724	0.5887	0.6909	0.5203	0.908	0.8621
SIDM00326	0.9911	0.9851	0.9911	0.9851	0.449	0.3728	0.6744	0.4435	0.9867	0.9762
SIDM00934	0.9227	0.8305	0.9227	0.8305	0.7984	0.6707	0.7096	0.4715	0.9227	0.8231
SIDM00105	0.9316	0.8462	0.9316	0.8462	0.7239	0.4916	0.6683	0.4435	0.928	0.8415
SIDM00088	0.9357	0.8313	0.9357	0.8313	0.7607	0.429	0.6797	0.46	0.9339	0.8306
SIDM00120	0.9396	0.717	0.9396	0.717	0.9181	0.657	0.6924	0.4602	0.9396	0.7212
SIDM00783	0.9806	0.9823	0.9806	0.9823	0.7241	0.7459	0.6628	0.4455	0.9796	0.9811
SIDM00789	0.9307	0.902	0.9307	0.902	0.7746	0.7353	0.6955	0.4716	0.9366	0.9101
SIDM00829	0.9385	0.9464	0.9385	0.9464	0.7181	0.7319	0.6901	0.4829	0.9376	0.9469
SIDM00136	0.952	0.9603	0.952	0.9603	0.9049	0.939	0.6837	0.4487	0.9461	0.9566
SIDM00791	0.8917	0.8551	0.8917	0.8551	0.6761	0.6184	0.6701	0.4613	0.8966	0.857
SIDM00139	0.9766	0.7	0.9766	0.7	0.4844	0.0435	0.6823	0.452	0.9766	0.7
SIDM00138	0.9604	0.8977	0.9604	0.8977	0.7635	0.5134	0.6901	0.4781	0.9508	0.8805
SIDM00116	0.9393	0.797	0.9393	0.797	0.6006	0.2969	0.6945	0.4717	0.9334	0.7713
SIDM00097	0.8638	0.7388	0.8638	0.7388	0.6362	0.3836	0.7119	0.4879	0.8617	0.7215
SIDM00148	0.8319	0.7763	0.8319	0.7763	0.6287	0.4451	0.6785	0.4648	0.817	0.7648
SIDM00125	0.9524	0.9253	0.9524	0.9253	0.3958	0.3553	0.6761	0.4672	0.9563	0.9347
SIDM00124	0.9249	0.8552	0.9249	0.8552	0.709	0.5968	0.6921	0.4774	0.9312	0.8604
SIDM00903	0.9486	0.9534	0.9486	0.9534	0.7833	0.8353	0.7364	0.5995	0.9431	0.95
SIDM00123	0.9125	0.8742	0.9125	0.8742	0.7442	0.7189	0.7375	0.6038	0.9173	0.8802
SIDM00122	0.8517	0.6905	0.8517	0.6905	0.5625	0.27	0.7248	0.5747	0.8505	0.6919
SIDM00121	0.9545	0.954	0.9545	0.954	0.5606	0.5583	0.7202	0.5753	0.9574	0.9581
SIDM00082	0.8858	0.7747	0.8858	0.7747	0.8746	0.7375	0.7118	0.5722	0.8903	0.7733
SIDM00135	0.9586	0.9577	0.9586	0.9577	0.6369	0.5838	0.7333	0.5829	0.9613	0.9598
SIDM00145	0.8872	0.83	0.8872	0.83	0.6188	0.5403	0.7371	0.5937	0.8919	0.8293
SIDM00079	0.894	0.6271	0.894	0.6271	0.7562	0.4551	0.7173	0.5603	0.8868	0.6302
SIDM00146	0.9276	0.9063	0.9276	0.9063	0.5676	0.4566	0.7251	0.5779	0.9219	0.8974
SIDM00826	0.8989	0.8719	0.8989	0.8719	0.818	0.8121	0.7034	0.5573	0.8925	0.867
SIDM00841	0.9435	0.901	0.9435	0.901	0.4528	0.367	0.7218	0.5469	0.937	0.8924
SIDM00087	0.945	0.9424	0.945	0.9424	0.7959	0.8143	0.715	0.5445	0.9469	0.9461
SIDM00117	0.8594	0.8562	0.8594	0.8562	0.7132	0.7647	0.6848	0.5249	0.8548	0.8548

Continued on next page

Table S8 – continued from previous page

Cell line	Ridge		Lasso		CNN		tCNN		BKDRP	
	AUC	AUPR	AUC	AUPR	AUC	AUPR	AUC	AUPR	AUC	AUPR
SIDM00151	0.9279	0.9541	0.9279	0.9541	0.7913	0.8876	0.6919	0.5267	0.9269	0.9545
SIDM00091	0.9133	0.8226	0.9133	0.8226	0.6878	0.511	0.6982	0.5293	0.9178	0.8309
SIDM00118	0.9908	0.9911	0.9908	0.9911	0.8834	0.9124	0.6988	0.5289	0.9908	0.9917
SIDM00133	0.915	0.7733	0.915	0.7733	0.8614	0.6765	0.7088	0.537	0.9163	0.7812
SIDM00149	0.9412	0.9503	0.9412	0.9503	0.8659	0.8952	0.6982	0.5339	0.9357	0.947
SIDM00107	0.9211	0.8974	0.9211	0.8974	0.8049	0.6837	0.7014	0.5288	0.906	0.875
SIDM00108	0.8571	0.7387	0.8571	0.7387	0.6951	0.475	0.7047	0.5324	0.8599	0.739
SIDM00094	0.9782	0.7798	0.9782	0.7798	0.8475	0.4812	0.701	0.5309	0.9782	0.7798
SIDM00113	0.8211	0.5803	0.8211	0.5803	0.4698	0.1255	0.7003	0.5334	0.8103	0.5612
SIDM00111	0.9652	0.912	0.9652	0.912	0.8439	0.7495	0.7064	0.5342	0.9616	0.9081
SIDM00083	0.9753	0.9775	0.9753	0.9775	0.5408	0.5412	0.7162	0.5378	0.9687	0.9708
SIDM00085	0.9813	0.9577	0.9813	0.9577	0.9363	0.8938	0.703	0.5276	0.9825	0.9602
SIDM00150	0.9375	0.9447	0.9375	0.9447	0.7362	0.7859	0.6976	0.5261	0.9283	0.9397
SIDM00092	0.8661	0.673	0.8661	0.673	0.7429	0.4731	0.7059	0.5354	0.8625	0.6651
SIDM00090	0.9624	0.9519	0.9624	0.9519	0.7143	0.7156	0.7092	0.5372	0.9605	0.9503
SIDM00132	0.9088	0.7918	0.9088	0.7918	0.8667	0.7361	0.6981	0.5293	0.9076	0.7835
SIDM00987	0.9449	0.933	0.9449	0.933	0.8414	0.867	0.694	0.5298	0.9449	0.9376
SIDM00490	0.9112	0.8302	0.9112	0.8302	0.8409	0.7743	0.7173	0.5469	0.9163	0.8333
SIDM01245	0.9012	0.9205	0.9012	0.9205	0.3905	0.5382	0.7104	0.5433	0.8975	0.9173
SIDM00850	0.9559	0.9601	0.9559	0.9601	0.7484	0.8039	0.6908	0.5307	0.9477	0.9542
SIDM00797	0.9402	0.9272	0.9402	0.9272	0.4634	0.4255	0.6954	0.5323	0.943	0.931
SIDM00795	0.9626	0.9547	0.9626	0.9547	0.5314	0.4729	0.7064	0.5385	0.9601	0.9481
SIDM00997	0.9462	0.9192	0.9462	0.9192	0.7906	0.6728	0.711	0.5404	0.9418	0.9111
SIDM00983	0.9317	0.8998	0.9317	0.8998	0.5938	0.3841	0.7217	0.5476	0.9375	0.9169
SIDM00999	0.9619	0.9217	0.9619	0.9217	0.4265	0.287	0.7009	0.5363	0.9556	0.912
SIDM00893	0.78	0.6163	0.7788	0.6147	0.5741	0.296	0.7018	0.5365	0.7753	0.605
SIDM00513	0.7894	0.7099	0.7894	0.7099	0.5885	0.5183	0.7249	0.5555	0.7856	0.7039
SIDM00506	0.9133	0.543	0.9133	0.543	0.29	0.0846	0.7073	0.5417	0.9067	0.5495
SIDM00954	0.8684	0.8341	0.8684	0.8341	0.7115	0.6126	0.691	0.5315	0.8665	0.834
SIDM00140	0.9674	0.9565	0.9674	0.9565	0.8583	0.881	0.7133	0.5457	0.9711	0.961
SIDM00956	0.963	0.6667	0.963	0.6667	0.3915	0.0581	0.7334	0.5493	0.9524	0.6465
SIDM00284	0.8654	0.7184	0.8654	0.7184	0.7451	0.5656	0.702	0.5322	0.8627	0.7268
SIDM00910	0.944	0.9464	0.944	0.9464	0.8026	0.8203	0.7047	0.5382	0.9449	0.9469
SIDM00130	0.9109	0.8117	0.9109	0.8117	0.7396	0.4956	0.7217	0.55	0.9028	0.7951
SIDM00923	0.9678	0.9567	0.9678	0.9567	0.8829	0.8556	0.7351	0.5527	0.9678	0.9564
SIDM00931	0.9702	0.9533	0.9702	0.9533	0.4544	0.4121	0.6946	0.5269	0.9722	0.9577
SIDM00890	0.8631	0.7659	0.8631	0.7659	0.526	0.2665	0.7091	0.5364	0.8529	0.7392
SIDM00886	0.8768	0.8993	0.8768	0.8993	0.4816	0.5607	0.6984	0.5285	0.8759	0.9012
SIDM00887	0.9883	0.9833	0.9883	0.9833	0.8439	0.8329	0.7161	0.5449	0.9873	0.9822
SIDM00888	0.932	0.9458	0.932	0.9458	0.9027	0.9158	0.7101	0.5358	0.9357	0.9481
SIDM00867	0.8475	0.6208	0.845	0.5732	0.5981	0.1357	0.7055	0.5302	0.8475	0.6228
SIDM00831	0.977	0.9842	0.977	0.9842	0.7207	0.7693	0.7008	0.536	0.9788	0.986
SIDM00947	0.9359	0.8645	0.9359	0.8645	0.7359	0.6329	0.7166	0.5413	0.9348	0.8568
SIDM00864	0.9155	0.8352	0.9155	0.8352	0.7905	0.6385	0.7152	0.5369	0.9167	0.8347
SIDM01001	0.8919	0.9249	0.8919	0.9249	0.6513	0.7321	0.6992	0.5351	0.8919	0.9238
SIDM01056	0.9191	0.8615	0.9191	0.8615	0.6319	0.4075	0.7209	0.5452	0.9213	0.8647
SIDM01047	0.8944	0.8903	0.8944	0.8903	0.5794	0.6083	0.7029	0.5426	0.8898	0.8822
SIDM01042	0.9111	0.9014	0.9111	0.9014	0.6171	0.5003	0.711	0.5378	0.912	0.9037
SIDM00860	0.8948	0.8905	0.8948	0.8905	0.4365	0.3728	0.7091	0.5368	0.8948	0.885
SIDM00213	0.9557	0.8881	0.9557	0.8881	0.5933	0.4478	0.6889	0.5212	0.9498	0.8759
SIDM00857	0.8888	0.8633	0.8888	0.8633	0.7737	0.7157	0.7022	0.5369	0.8868	0.8616
SIDM01055	0.9717	0.9633	0.9717	0.9633	0.4224	0.4118	0.6961	0.5315	0.9707	0.9618
SIDM00274	0.9485	0.9113	0.9485	0.9113	0.785	0.6622	0.6844	0.5258	0.9462	0.9055
SIDM01057	0.8423	0.9201	0.8423	0.9201	0.8021	0.9002	0.7059	0.5346	0.8402	0.9186
SIDM00273	0.9877	0.9832	0.9877	0.9832	0.8585	0.8023	0.7023	0.5332	0.9877	0.9829

Continued on next page

Table S8 – continued from previous page

Cell line	Ridge		Lasso		CNN		tCNN		BKDRP	
	AUC	AUPR	AUC	AUPR	AUC	AUPR	AUC	AUPR	AUC	AUPR
SIDM00227	0.9616	0.9005	0.9616	0.9005	0.8451	0.7002	0.7113	0.5451	0.9616	0.9016
SIDM00544	0.9246	0.8604	0.9246	0.8604	0.8419	0.7522	0.7098	0.5405	0.9205	0.8487
SIDM01259	0.9201	0.9245	0.9201	0.9245	0.6171	0.6119	0.7117	0.543	0.9238	0.9288
SIDM00852	0.9278	0.9306	0.9278	0.9306	0.8657	0.8548	0.691	0.5234	0.9269	0.9294
SIDM00882	0.7662	0.4902	0.7662	0.4902	0.7179	0.4075	0.7122	0.5454	0.7587	0.4868
SIDM00673	0.9093	0.7836	0.9093	0.7836	0.568	0.3209	0.6911	0.5254	0.9036	0.7708
SIDM00596	0.9831	0.8857	0.9831	0.8857	0.9274	0.7063	0.7219	0.5443	0.9831	0.8753
SIDM00290	0.9507	0.9528	0.9507	0.9528	0.7021	0.672	0.707	0.5308	0.9393	0.9425
SIDM00064	0.9549	0.893	0.9549	0.893	0.7002	0.477	0.7146	0.5379	0.9525	0.8878
SIDM00291	0.9454	0.9264	0.9454	0.9264	0.7551	0.635	0.6961	0.5259	0.9366	0.9199
SIDM00589	0.9522	0.9573	0.9522	0.9573	0.7098	0.7178	0.7173	0.5381	0.9559	0.9588
SIDM00588	0.8564	0.802	0.8564	0.802	0.726	0.6326	0.7059	0.5413	0.8564	0.8092
SIDM00676	0.88	0.7089	0.88	0.7089	0.7629	0.4888	0.718	0.5469	0.88	0.7033
SIDM00678	0.8889	0.6856	0.8889	0.6856	0.7593	0.2551	0.7159	0.543	0.8852	0.6846
SIDM00695	0.9444	0.9216	0.9444	0.9216	0.7134	0.7071	0.7096	0.5428	0.9426	0.9178
SIDM00679	0.8791	0.6719	0.8791	0.6719	0.6082	0.2208	0.7024	0.5374	0.8713	0.6623
SIDM00585	0.9159	0.6466	0.9159	0.6466	0.6336	0.183	0.6999	0.5295	0.9289	0.7161
SIDM01060	0.8985	0.8514	0.8985	0.8514	0.7541	0.624	0.7045	0.5337	0.8995	0.8479
SIDM01206	0.9229	0.9273	0.9229	0.9273	0.58	0.6194	0.716	0.5428	0.9333	0.9396
SIDM00225	0.9361	0.9012	0.9361	0.9012	0.5986	0.524	0.7098	0.5384	0.932	0.8972
SIDM00224	0.9182	0.8106	0.9182	0.8106	0.7377	0.6058	0.7126	0.54	0.9213	0.8236
SIDM01012	0.9459	0.9732	0.9459	0.9732	0.5928	0.7051	0.7063	0.5405	0.9406	0.9702
SIDM00613	0.9697	0.9333	0.9697	0.9333	0.7294	0.6334	0.7331	0.5704	0.9686	0.937
SIDM01007	0.8959	0.8805	0.8959	0.8805	0.7908	0.7707	0.7185	0.5682	0.9005	0.8827
SIDM01008	0.9384	0.9445	0.9384	0.9445	0.4017	0.4945	0.7324	0.572	0.9347	0.941
SIDM00687	0.761	0.5621	0.761	0.5621	0.581	0.3546	0.7233	0.5663	0.7514	0.5436
SIDM01003	0.9376	0.9596	0.9376	0.9596	0.6852	0.7537	0.7304	0.5686	0.9444	0.9633
SIDM00785	0.8915	0.8361	0.8915	0.8361	0.5899	0.4452	0.7117	0.5532	0.8926	0.8362
SIDM00612	0.9157	0.6797	0.9157	0.6797	0.7669	0.5114	0.7185	0.565	0.919	0.6723
SIDM01019	0.963	0.966	0.963	0.966	0.7472	0.7324	0.7176	0.5649	0.9583	0.9607
SIDM01020	0.939	0.9563	0.939	0.9563	0.6562	0.7185	0.7221	0.5679	0.939	0.9559
SIDM00533	0.9203	0.8353	0.9203	0.8353	0.7651	0.6148	0.7313	0.5738	0.9176	0.8441
SIDM00531	0.9059	0.7818	0.9059	0.7818	0.7793	0.5552	0.7146	0.5666	0.912	0.7895
SIDM00541	0.904	0.8974	0.904	0.8974	0.7782	0.7663	0.7317	0.5752	0.8966	0.8905
SIDM00537	0.9553	0.9487	0.9553	0.9487	0.9217	0.9287	0.7256	0.5703	0.9562	0.9521
SIDM00523	0.7875	0.5809	0.7875	0.5809	0.5214	0.2152	0.7083	0.5524	0.7893	0.5984
SIDM00553	0.9153	0.9355	0.9153	0.9355	0.6246	0.6732	0.7106	0.5638	0.9241	0.9404
SIDM00308	0.9307	0.9539	0.9307	0.9539	0.642	0.7717	0.7309	0.573	0.9307	0.9535
SIDM01033	0.9676	0.9608	0.9676	0.9608	0.6769	0.7037	0.7178	0.5642	0.9639	0.9582
SIDM00555	0.9824	0.9714	0.9824	0.9714	0.7159	0.634	0.7331	0.5734	0.9814	0.9672
SIDM00556	0.9073	0.8123	0.9061	0.7956	0.5902	0.4002	0.7256	0.5656	0.9098	0.8217
SIDM01031	0.9667	0.9666	0.9667	0.9666	0.6253	0.5651	0.7353	0.5728	0.9653	0.9647
SIDM01030	0.9635	0.9454	0.9635	0.9454	0.9221	0.9039	0.7221	0.5684	0.9644	0.9466
SIDM01029	0.9354	0.886	0.9354	0.886	0.5196	0.4542	0.7299	0.5748	0.9397	0.8938
SIDM01027	0.8696	0.8282	0.8696	0.8282	0.6698	0.5571	0.7192	0.5645	0.8758	0.8305
SIDM00346	0.9297	0.9481	0.9297	0.9481	0.698	0.8022	0.7239	0.5695	0.9326	0.9518
SIDM00549	0.8454	0.8737	0.8454	0.8737	0.5683	0.5887	0.7245	0.5698	0.8406	0.8693
SIDM00683	0.953	0.9499	0.953	0.9499	0.7991	0.8179	0.7137	0.5664	0.9429	0.9396
SIDM00342	0.9017	0.9153	0.9017	0.9153	0.6149	0.6239	0.7387	0.5775	0.909	0.918
SIDM00839	0.9373	0.9496	0.9373	0.9496	0.7696	0.8499	0.7242	0.5611	0.9392	0.9508
SIDM00776	0.9203	0.7181	0.9203	0.7181	0.8173	0.5329	0.7178	0.566	0.9135	0.7097
SIDM00660	0.9326	0.9121	0.9326	0.9121	0.8433	0.8316	0.7377	0.5786	0.924	0.9016
SIDM00633	0.8051	0.7623	0.8051	0.7623	0.7047	0.6367	0.7152	0.5614	0.8039	0.7589
SIDM00528	0.9457	0.9077	0.9457	0.9077	0.6354	0.4688	0.7425	0.5798	0.9387	0.8938
SIDM00272	0.8987	0.8655	0.8987	0.8655	0.6299	0.6006	0.7232	0.5654	0.9059	0.8706

Continued on next page

Table S8 – continued from previous page

Cell line	Ridge		Lasso		CNN		tCNN		BKDRP	
	AUC	AUPR	AUC	AUPR	AUC	AUPR	AUC	AUPR	AUC	AUPR
SIDM00628	0.9402	0.9391	0.9402	0.9391	0.9009	0.8873	0.7268	0.5726	0.9402	0.9426
SIDM00336	0.926	0.7841	0.926	0.7841	0.8737	0.7538	0.7106	0.5599	0.9202	0.7854
SIDM00526	0.8879	0.8868	0.8879	0.8868	0.739	0.7639	0.724	0.572	0.8879	0.889
SIDM00545	0.9375	0.834	0.9375	0.834	0.6326	0.3113	0.735	0.574	0.928	0.8084
SIDM00335	0.8963	0.7986	0.8963	0.7986	0.7763	0.6487	0.7304	0.5771	0.8975	0.8061
SIDM00334	0.9661	0.8806	0.9661	0.8806	0.8125	0.5798	0.7256	0.568	0.9607	0.8632
SIDM00260	0.958	0.8886	0.958	0.8886	0.7131	0.4562	0.7238	0.5615	0.9604	0.8943
SIDM00441	0.9141	0.9545	0.9141	0.9545	0.5785	0.715	0.7109	0.5605	0.9141	0.9553
SIDM00532	0.9597	0.9315	0.9597	0.9315	0.6208	0.5474	0.7296	0.5758	0.9542	0.9141
SIDM00504	0.9476	0.95	0.9476	0.95	0.6475	0.6052	0.7222	0.5639	0.9374	0.9425
SIDM00433	0.8916	0.8894	0.8916	0.8894	0.7732	0.773	0.728	0.5688	0.8916	0.8908
SIDM00431	0.906	0.9362	0.906	0.9362	0.6857	0.7571	0.7341	0.5743	0.9117	0.9367
SIDM01023	0.9667	0.9834	0.9667	0.9834	0.9486	0.9751	0.7138	0.5565	0.9667	0.9836
SIDM00222	0.9653	0.9232	0.9653	0.9232	0.7928	0.705	0.7359	0.577	0.9597	0.909
SIDM00562	0.9278	0.8883	0.9278	0.8883	0.7093	0.6178	0.7318	0.5737	0.919	0.8856
SIDM00244	0.9126	0.9399	0.9126	0.9399	0.6504	0.6705	0.7461	0.5788	0.907	0.9363
SIDM01130	0.9606	0.9535	0.9606	0.9535	0.7125	0.6365	0.7159	0.5666	0.9596	0.9508
SIDM01146	0.8977	0.8352	0.8977	0.8352	0.4948	0.3472	0.7293	0.5731	0.8977	0.8341
SIDM01144	0.9211	0.9095	0.9211	0.9095	0.7284	0.7223	0.7353	0.5724	0.9239	0.9168
SIDM01145	0.9413	0.9642	0.9413	0.9642	0.5349	0.6624	0.7194	0.573	0.9429	0.9657
SIDM00577	0.9316	0.9289	0.9316	0.9289	0.7009	0.6327	0.7161	0.5678	0.9326	0.9283
SIDM00576	0.9265	0.9545	0.9265	0.9545	0.5388	0.6402	0.7297	0.5736	0.9291	0.9536
SIDM00580	0.9605	0.9573	0.9605	0.9573	0.7868	0.7679	0.7378	0.5725	0.9577	0.956
SIDM00572	0.9023	0.8926	0.9023	0.8926	0.5771	0.5961	0.7207	0.5664	0.8947	0.8849
SIDM00778	0.9143	0.8467	0.9143	0.8467	0.7841	0.6167	0.7159	0.5659	0.9112	0.8462
SIDM00779	0.8472	0.7538	0.8472	0.7538	0.6835	0.545	0.7316	0.5725	0.8482	0.7694
SIDM00925	0.9032	0.4507	0.9032	0.4507	0.8024	0.292	0.7082	0.5602	0.9032	0.4521
SIDM01147	0.8257	0.7722	0.8257	0.7722	0.7568	0.6415	0.7166	0.5627	0.8284	0.7787
SIDM00722	0.9225	0.8645	0.9225	0.8645	0.8889	0.8362	0.7255	0.5691	0.9181	0.8514
SIDM00755	0.8547	0.5474	0.8547	0.5474	0.707	0.2775	0.7253	0.5626	0.8547	0.5462
SIDM00718	0.9397	0.8999	0.9397	0.8999	0.872	0.7914	0.7325	0.5743	0.9333	0.8909
SIDM00720	0.8986	0.6821	0.8986	0.6821	0.6491	0.4087	0.7408	0.5775	0.883	0.6679
SIDM00749	0.8484	0.5235	0.8484	0.5235	0.5248	0.1492	0.7165	0.5597	0.8542	0.5274
SIDM00743	0.8339	0.7213	0.8339	0.7213	0.7249	0.6534	0.7172	0.5653	0.836	0.7265
SIDM00738	0.9394	0.9323	0.9394	0.9323	0.836	0.8053	0.7325	0.5657	0.9338	0.9239
SIDM00758	0.8825	0.8441	0.8825	0.8441	0.7773	0.7669	0.7251	0.5657	0.8816	0.8437
SIDM01129	0.9712	0.9577	0.9712	0.9577	0.7904	0.7088	0.7392	0.5812	0.9712	0.9574
SIDM00567	0.95	0.9084	0.95	0.9084	0.6833	0.5352	0.7275	0.5694	0.9467	0.8972
SIDM00457	0.94	0.943	0.94	0.943	0.7	0.6775	0.7284	0.5691	0.9362	0.9374
SIDM00239	0.9232	0.7872	0.9232	0.7872	0.725	0.3853	0.7335	0.5771	0.9214	0.7769
SIDM00463	0.93	0.9605	0.93	0.9605	0.5014	0.6194	0.716	0.5622	0.93	0.9593
SIDM00364	0.9109	0.9218	0.9109	0.9218	0.596	0.6169	0.7225	0.5618	0.9091	0.919
SIDM00363	0.926	0.9354	0.926	0.9354	0.6824	0.6538	0.7356	0.5796	0.926	0.9331
SIDM00241	0.9027	0.9091	0.9027	0.9091	0.7383	0.723	0.7385	0.5813	0.9008	0.9091
SIDM00220	0.9615	0.9617	0.9615	0.9617	0.561	0.5383	0.7347	0.5795	0.9589	0.9589
SIDM00451	0.9404	0.9574	0.9404	0.9574	0.7046	0.7583	0.7187	0.5606	0.9413	0.9581
SIDM00359	0.8695	0.6585	0.8695	0.6585	0.7761	0.5707	0.7378	0.5792	0.8695	0.6577
SIDM01092	0.9513	0.9367	0.9513	0.9367	0.6312	0.5125	0.7139	0.5641	0.9491	0.9329
SIDM01090	0.9649	0.955	0.9649	0.955	0.7635	0.7333	0.7384	0.5792	0.9582	0.9464
SIDM00403	0.9063	0.9368	0.9049	0.9365	0.5462	0.6113	0.7137	0.5632	0.909	0.9386
SIDM01086	0.8977	0.9093	0.8977	0.9093	0.5672	0.5821	0.7253	0.5725	0.9138	0.9208
SIDM00402	0.9666	0.9461	0.9666	0.9461	0.8231	0.7226	0.7303	0.5792	0.9596	0.9354
SIDM01080	0.9293	0.8577	0.9293	0.8577	0.7935	0.7811	0.7189	0.5669	0.9315	0.8555
SIDM01079	0.9185	0.8686	0.9185	0.8686	0.8685	0.8245	0.7153	0.5635	0.913	0.8553
SIDM01096	0.8869	0.8414	0.8869	0.8414	0.7739	0.7041	0.7308	0.5693	0.886	0.8478

Continued on next page

Table S8 – continued from previous page

Cell line	Ridge		Lasso		CNN		tCNN		BKDRP	
	AUC	AUPR	AUC	AUPR	AUC	AUPR	AUC	AUPR	AUC	AUPR
SIDM01110	0.9796	0.9538	0.9796	0.9538	0.8247	0.6926	0.7164	0.5657	0.9844	0.965
SIDM01108	0.8513	0.5117	0.8513	0.5117	0.5364	0.1895	0.7199	0.5623	0.8542	0.5579
SIDM01107	0.7835	0.5594	0.7835	0.5594	0.3721	0.1914	0.7199	0.5672	0.7674	0.5694
SIDM01106	0.9043	0.8121	0.9043	0.8121	0.7707	0.6148	0.7325	0.5752	0.9054	0.8137
SIDM01103	0.9586	0.8836	0.9586	0.8836	0.8219	0.7141	0.7365	0.5793	0.9563	0.8835
SIDM00379	0.9545	0.8814	0.9545	0.8814	0.8328	0.7243	0.7242	0.566	0.9513	0.8724
SIDM00373	0.9752	0.9597	0.9752	0.9597	0.7407	0.5932	0.7311	0.579	0.9721	0.9536
SIDM01180	0.902	0.7836	0.902	0.7836	0.7765	0.5806	0.7211	0.57	0.8928	0.7696
SIDM01199	0.9653	0.9289	0.9653	0.9289	0.7593	0.5571	0.7262	0.5748	0.9699	0.9359
SIDM01194	0.9408	0.8352	0.9408	0.8352	0.4978	0.1744	0.7433	0.5775	0.9364	0.8404
SIDM00781	0.9513	0.8764	0.9513	0.8764	0.8488	0.666	0.7289	0.5703	0.9525	0.876
SIDM01154	0.9421	0.9509	0.9412	0.9505	0.7454	0.7951	0.7247	0.576	0.9393	0.9474
SIDM00814	0.9309	0.9367	0.9309	0.9367	0.8931	0.8826	0.7017	0.5108	0.9429	0.9463
SIDM01155	0.8909	0.8191	0.8909	0.8191	0.7817	0.7642	0.7068	0.5257	0.8919	0.8126
SIDM00835	0.8772	0.5447	0.8772	0.5447	0.7047	0.2298	0.7105	0.5204	0.8772	0.555
SIDM00812	0.7875	0.4764	0.7875	0.4764	0.7232	0.3123	0.7091	0.5235	0.7875	0.5148
SIDM00834	0.8447	0.6432	0.8447	0.6432	0.7213	0.3862	0.7034	0.5099	0.8374	0.6383
SIDM00420	0.9438	0.8506	0.9438	0.8506	0.7363	0.5306	0.7038	0.5155	0.9463	0.8593
SIDM01164	0.9723	0.9691	0.9723	0.9691	0.5481	0.4783	0.7059	0.5117	0.9736	0.9701
SIDM00837	0.8873	0.9093	0.8873	0.9093	0.7755	0.7982	0.7062	0.5206	0.8902	0.9089
SIDM01168	0.8636	0.6954	0.8636	0.6954	0.6488	0.4618	0.72	0.535	0.8621	0.6929
SIDM00833	0.9121	0.8104	0.9121	0.8104	0.8104	0.6176	0.6937	0.5143	0.908	0.8017
SIDM00832	0.9073	0.8225	0.9073	0.8225	0.84	0.7499	0.704	0.515	0.9063	0.8194
SIDM00782	0.9363	0.8645	0.9363	0.8645	0.82	0.5104	0.7107	0.5161	0.9313	0.8711
SIDM00251	0.9551	0.9318	0.9551	0.9318	0.6051	0.4422	0.7155	0.5159	0.9513	0.9264
SIDM01205	0.9676	0.9671	0.9676	0.9671	0.7491	0.7449	0.6972	0.5131	0.963	0.963
SIDM00317	0.9788	0.7222	0.9788	0.7222	0.8942	0.3207	0.7006	0.5117	0.9788	0.8095
SIDM01000	0.8924	0.8223	0.8924	0.8223	0.7211	0.6514	0.7142	0.5165	0.8912	0.8234
SIDM00411	0.9492	0.6258	0.9492	0.6258	0.6804	0.1772	0.7052	0.5134	0.954	0.6521
SIDM00316	0.9767	0.969	0.9767	0.969	0.6542	0.5108	0.696	0.513	0.9788	0.9691
SIDM00971	0.6653	0.3029	0.6653	0.3029	0.5445	0.1348	0.7114	0.5185	0.6419	0.2886
SIDM00235	0.8497	0.73	0.8497	0.73	0.6797	0.4432	0.7038	0.5178	0.8523	0.7332
SIDM00479	0.9173	0.8831	0.9173	0.8831	0.7894	0.711	0.7153	0.5218	0.9115	0.8694
SIDM00469	0.9069	0.8399	0.9069	0.8399	0.7683	0.6981	0.7067	0.5142	0.9026	0.8321
SIDM00480	0.9231	0.8091	0.9231	0.8091	0.6816	0.4982	0.6929	0.5059	0.9356	0.8239
SIDM00477	0.9484	0.9172	0.9484	0.9172	0.8026	0.7404	0.7163	0.5272	0.9464	0.9173
SIDM00282	0.9037	0.8512	0.9037	0.8512	0.6667	0.4402	0.7121	0.518	0.909	0.8606
SIDM01248	0.9048	0.7632	0.9048	0.7632	0.6633	0.3783	0.6964	0.5139	0.8963	0.7532
SIDM00842	0.8733	0.8615	0.8733	0.8615	0.7651	0.692	0.7105	0.5255	0.877	0.8661
SIDM00233	0.9274	0.9408	0.9274	0.9408	0.7684	0.7781	0.7042	0.5238	0.9246	0.9397
SIDM00957	0.859	0.5267	0.859	0.5267	0.6262	0.1414	0.7017	0.5175	0.8525	0.589
SIDM00142	0.9351	0.8426	0.9351	0.8426	0.7565	0.6327	0.7124	0.5222	0.9318	0.8347
SIDM00143	0.8843	0.8013	0.8843	0.8013	0.688	0.5703	0.7017	0.5175	0.8822	0.8173
SIDM00343	0.9589	0.8114	0.9589	0.8114	0.7929	0.5939	0.692	0.5058	0.9679	0.8499
SIDM00988	0.9506	0.9452	0.9506	0.9452	0.671	0.6083	0.7112	0.5198	0.9525	0.9482
SIDM01233	0.9675	0.9171	0.9675	0.9171	0.7813	0.5934	0.7159	0.5266	0.96	0.9093
SIDM00899	1	1	1	1	0.7077	0.05	0.7229	0.5273	1	1
SIDM00898	0.909	0.9122	0.909	0.9122	0.7045	0.6161	0.6982	0.521	0.9072	0.9059
SIDM00962	0.8868	0.934	0.8855	0.9335	0.55	0.7104	0.7131	0.5211	0.8908	0.9357
SIDM00324	0.9335	0.9313	0.9335	0.9313	0.7588	0.6899	0.6971	0.5112	0.9307	0.9227
SIDM00901	0.8969	0.8283	0.8969	0.8283	0.7472	0.6531	0.6952	0.5158	0.8959	0.8309
SIDM01237	0.9214	0.6625	0.9196	0.6292	0.8	0.4427	0.7092	0.5206	0.9143	0.6746
SIDM01228	0.925	0.8772	0.925	0.8772	0.8076	0.6866	0.7265	0.5387	0.9207	0.8622
SIDM00744	0.8848	0.7893	0.8848	0.7893	0.6255	0.4826	0.7097	0.527	0.88	0.7863
SIDM01087	0.9537	0.9586	0.9537	0.9586	0.7639	0.7803	0.7254	0.5299	0.9593	0.9636

Continued on next page

Table S8 – continued from previous page

Cell line	Ridge		Lasso		CNN		tCNN		BKDRP	
	AUC	AUPR	AUC	AUPR	AUC	AUPR	AUC	AUPR	AUC	AUPR
SIDM01081	0.9563	0.9234	0.9563	0.9234	0.7928	0.7255	0.7151	0.5277	0.9541	0.9179
SIDM01197	0.8241	0.6671	0.8229	0.6652	0.7731	0.6303	0.719	0.5259	0.8206	0.6628
SIDM01165	0.9413	0.7127	0.9413	0.7127	0.8112	0.4957	0.7142	0.5292	0.9388	0.7071
SIDM01187	0.8578	0.8008	0.8578	0.8008	0.5359	0.3341	0.6886	0.5116	0.866	0.8005
SIDM01083	0.9172	0.8724	0.9172	0.8724	0.7119	0.4963	0.7052	0.5214	0.9076	0.8468
SIDM00943	0.8775	0.7474	0.8775	0.7474	0.7668	0.5204	0.7102	0.5169	0.8755	0.7557
SIDM00937	0.9449	0.9492	0.9449	0.9492	0.6903	0.7149	0.709	0.5212	0.9384	0.9432
SIDM00896	0.924	0.95	0.922	0.9492	0.6491	0.6842	0.7012	0.5171	0.9113	0.9416
SIDM00849	0.9225	0.8562	0.9225	0.8562	0.6655	0.5252	0.7108	0.5286	0.9225	0.8542
SIDM00803	0.9161	0.8689	0.9161	0.8689	0.7776	0.6926	0.7068	0.5214	0.9102	0.8672
SIDM00980	0.9785	0.9652	0.9785	0.9652	0.8234	0.7955	0.7046	0.5169	0.9746	0.9612
SIDM00989	0.9394	0.936	0.9394	0.936	0.8164	0.8144	0.7224	0.5346	0.9459	0.9417
SIDM00933	0.9519	0.9386	0.9519	0.9386	0.4855	0.4952	0.7148	0.5307	0.951	0.938
SIDM00384	0.9449	0.9472	0.9449	0.9472	0.7365	0.7858	0.7029	0.5178	0.9467	0.9491
SIDM00543	0.9302	0.8599	0.9302	0.8599	0.7462	0.6822	0.706	0.5175	0.9262	0.8551
SIDM00492	0.8865	0.8594	0.8865	0.8594	0.7481	0.7553	0.7188	0.525	0.8846	0.857
SIDM00502	0.939	0.9602	0.939	0.9602	0.6341	0.7235	0.6917	0.5098	0.9299	0.9552
SIDM00495	0.8478	0.9147	0.8478	0.9147	0.5352	0.7049	0.6932	0.5054	0.8427	0.9089
SIDM00540	0.8468	0.871	0.8468	0.871	0.7378	0.8173	0.7172	0.5251	0.8468	0.8739
SIDM00511	0.9431	0.9483	0.9431	0.9483	0.9378	0.9478	0.7113	0.5208	0.9484	0.9562
SIDM00212	0.9519	0.9054	0.9519	0.9054	0.5652	0.3807	0.6967	0.5149	0.9588	0.9147
SIDM00211	0.8737	0.9164	0.8737	0.9164	0.7255	0.8081	0.7093	0.5295	0.8718	0.9129
SIDM00446	0.8929	0.9231	0.8929	0.9231	0.5985	0.6799	0.7054	0.5203	0.8967	0.9226
SIDM00332	0.913	0.8382	0.913	0.8382	0.6201	0.3915	0.7219	0.5298	0.9048	0.821
SIDM00958	0.8472	0.8735	0.8472	0.8735	0.5937	0.6495	0.7007	0.5143	0.8397	0.8689
SIDM00932	0.8507	0.8606	0.8507	0.8606	0.7475	0.7272	0.7249	0.5358	0.8544	0.8605
SIDM01052	0.9476	0.6013	0.9476	0.6013	0.8427	0.2835	0.7132	0.5229	0.9597	0.7049
SIDM00681	0.8837	0.6947	0.8837	0.6947	0.4797	0.2342	0.7162	0.5248	0.8889	0.7323
SIDM00499	0.9	0.813	0.9	0.813	0.7511	0.6392	0.7031	0.5205	0.9	0.8157
SIDM00996	0.9652	0.9142	0.9652	0.9142	0.7563	0.5072	0.7159	0.5221	0.9616	0.9072
SIDM00992	0.9554	0.9387	0.9554	0.9387	0.756	0.6533	0.704	0.515	0.9573	0.9374
SIDM00938	0.9236	0.9022	0.9236	0.9022	0.6232	0.6225	0.7033	0.5176	0.9236	0.9022
SIDM00935	0.9153	0.9558	0.9153	0.9558	0.7624	0.8845	0.7135	0.5177	0.9184	0.9571
SIDM00488	0.9776	0.9643	0.9776	0.9643	0.8185	0.7801	0.6971	0.5122	0.9785	0.9662
SIDM00592	0.976	0.9723	0.976	0.9723	0.7834	0.8069	0.7074	0.5211	0.9751	0.9713
SIDM00496	0.9153	0.8738	0.9153	0.8738	0.7704	0.6652	0.6965	0.5153	0.9164	0.8725
SIDM00686	0.9306	0.7954	0.9306	0.7954	0.5041	0.1779	0.7069	0.5138	0.9322	0.7966
SIDM00056	0.9486	0.9433	0.9486	0.9433	0.6933	0.7572	0.7201	0.5346	0.9541	0.952
SIDM00312	0.9317	0.9438	0.9317	0.9438	0.8302	0.8683	0.7073	0.5188	0.9307	0.9413
SIDM00630	0.9039	0.8184	0.9039	0.8184	0.7717	0.7081	0.6989	0.5152	0.905	0.8226
SIDM00759	0.9189	0.9142	0.9189	0.9142	0.7829	0.794	0.709	0.5205	0.9236	0.9222
SIDM00560	0.9259	0.7854	0.9259	0.7854	0.5648	0.2864	0.7027	0.5198	0.9241	0.7581
SIDM00529	0.9644	0.9342	0.9644	0.9342	0.4687	0.218	0.7064	0.5137	0.9601	0.921
SIDM00247	0.8669	0.7789	0.8669	0.7789	0.8345	0.642	0.7101	0.5218	0.8692	0.7836
SIDM01138	0.9192	0.8054	0.9192	0.8054	0.6982	0.4726	0.714	0.5207	0.9162	0.8028
SIDM01136	0.9446	0.8355	0.9446	0.8355	0.7429	0.5668	0.7104	0.5275	0.9339	0.832
SIDM01135	0.9124	0.7719	0.9124	0.7719	0.7124	0.3993	0.7131	0.5261	0.9176	0.7829
SIDM00218	0.9279	0.5597	0.9279	0.5597	0.7049	0.3415	0.6873	0.4568	0.9279	0.6209
SIDM01093	0.9634	0.8772	0.9634	0.8772	0.8103	0.4051	0.7116	0.4708	0.9677	0.8672
SIDM00381	0.9565	0.8834	0.9565	0.8834	0.7261	0.6607	0.6971	0.4507	0.9587	0.8869
SIDM00484	0.9467	0.6327	0.9467	0.6327	0.3633	0.0952	0.6973	0.4605	0.94	0.6148
SIDM00325	0.9375	0.917	0.9375	0.917	0.8275	0.7074	0.6934	0.4478	0.9375	0.917
SIDM00328	0.8196	0.7755	0.8196	0.7755	0.6344	0.5982	0.705	0.457	0.8196	0.7829
SIDM00410	0.9519	0.9582	0.9519	0.9582	0.5398	0.5078	0.7203	0.4746	0.9533	0.9596
SIDM00963	0.8094	0.6541	0.8094	0.6541	0.5176	0.2632	0.7018	0.4482	0.8118	0.6579

Continued on next page

Table S8 – continued from previous page

Cell line	Ridge		Lasso		CNN		tCNN		BKDRP	
	AUC	AUPR	AUC	AUPR	AUC	AUPR	AUC	AUPR	AUC	AUPR
SIDM00481	0.9275	0.8248	0.9275	0.8248	0.6991	0.4133	0.7133	0.4678	0.9306	0.8249
SIDM00732	0.9556	0.921	0.9556	0.921	0.7503	0.6109	0.6983	0.4605	0.963	0.9332
SIDM00542	0.9464	0.8746	0.9464	0.8746	0.7404	0.5172	0.7	0.4566	0.9505	0.875
SIDM00920	0.9275	0.8478	0.9275	0.8478	0.5507	0.3626	0.7138	0.4573	0.9286	0.8592
SIDM00918	0.8813	0.782	0.8813	0.782	0.4281	0.2511	0.6959	0.4522	0.8719	0.7617
SIDM00892	1	1	1	1	0.2545	0.0238	0.7123	0.4672	1	1
SIDM00659	0.9586	0.9438	0.9586	0.9438	0.705	0.6181	0.7057	0.4615	0.9586	0.9425
SIDM00289	0.858	0.8676	0.858	0.8676	0.5312	0.528	0.6911	0.4506	0.858	0.8676
SIDM00156	0.8993	0.8075	0.8993	0.8075	0.485	0.2362	0.6935	0.449	0.8915	0.7997
SIDM00257	0.9026	0.9028	0.9016	0.9023	0.6542	0.6159	0.7141	0.4678	0.8966	0.9005
SIDM00256	0.9194	0.9368	0.9194	0.9368	0.6015	0.6401	0.697	0.4557	0.9298	0.9437
SIDM00578	0.9565	0.9519	0.9565	0.9519	0.4923	0.4771	0.7116	0.4684	0.9565	0.9508
SIDM01016	0.9191	0.9329	0.9191	0.9329	0.6893	0.6792	0.7044	0.4646	0.9182	0.9335
SIDM00680	0.9205	0.8415	0.9205	0.8415	0.7552	0.5956	0.7028	0.4633	0.9153	0.8355
SIDM00485	0.9524	0.9334	0.9524	0.9334	0.8254	0.7718	0.6965	0.4521	0.9563	0.9384
SIDM00501	0.9675	0.8816	0.9675	0.8816	0.8062	0.6113	0.6953	0.4546	0.9663	0.8779
SIDM00487	0.8963	0.89	0.8963	0.89	0.7	0.684	0.7017	0.4666	0.8944	0.8889
SIDM01045	0.8627	0.7184	0.8627	0.7184	0.6928	0.4398	0.7123	0.4575	0.8588	0.7048
SIDM01040	0.9326	0.8035	0.9326	0.8035	0.4326	0.1665	0.7047	0.4627	0.937	0.8116
SIDM00101	0.8862	0.506	0.8862	0.506	0.8547	0.3577	0.7114	0.4634	0.8935	0.5082
SIDM00100	0.9677	0.6778	0.9677	0.6778	0.7661	0.555	0.7108	0.4544	0.9597	0.6576
SIDM00098	0.9566	0.9165	0.9566	0.9165	0.8032	0.6239	0.7005	0.4549	0.9492	0.9025
SIDM00114	0.9254	0.6407	0.9254	0.6407	0.6711	0.3212	0.7028	0.4633	0.9123	0.6308
SIDM00598	0.9715	0.9697	0.9715	0.9697	0.8163	0.8322	0.6972	0.4498	0.9697	0.9683
SIDM01067	0.8032	0.7437	0.8032	0.7437	0.6159	0.4906	0.7102	0.4602	0.8169	0.7661
SIDM00665	0.9005	0.8365	0.9005	0.8365	0.8392	0.7923	0.6979	0.4706	0.8984	0.8346
SIDM01066	0.9232	0.8916	0.9232	0.8916	0.7786	0.6925	0.6968	0.456	0.9201	0.8914
SIDM00600	0.944	0.9439	0.944	0.9439	0.7208	0.7733	0.7015	0.4555	0.9385	0.9401
SIDM00602	0.9484	0.9235	0.9484	0.9235	0.8214	0.7907	0.6938	0.4465	0.9454	0.9146
SIDM00617	0.9194	0.8517	0.9194	0.8517	0.6488	0.5384	0.6958	0.4518	0.9143	0.8276
SIDM00616	0.9363	0.8342	0.9363	0.8342	0.6782	0.5034	0.7023	0.4502	0.9352	0.8297
SIDM00615	0.9434	0.8252	0.9434	0.8252	0.7431	0.5523	0.6951	0.4521	0.9463	0.8296
SIDM00614	0.8772	0.5504	0.8772	0.5504	0.6121	0.3519	0.7009	0.4531	0.8752	0.5569
SIDM00127	0.8393	0.8043	0.8393	0.8043	0.6124	0.5375	0.7048	0.4531	0.8491	0.816
SIDM00551	0.9525	0.9466	0.9525	0.9466	0.9282	0.9126	0.691	0.4547	0.945	0.9414
SIDM00685	0.913	0.8406	0.913	0.8406	0.7098	0.5315	0.7099	0.4606	0.9152	0.8482
SIDM00684	0.9472	0.6856	0.9472	0.6856	0.8722	0.3871	0.7075	0.4626	0.95	0.7411
SIDM00629	0.9885	0.9192	0.9885	0.9192	0.5881	0.2451	0.7067	0.4582	0.9847	0.8871
SIDM00527	0.9676	0.9729	0.9676	0.9729	0.5706	0.6383	0.6912	0.4568	0.9686	0.9745
SIDM00333	0.9566	0.8968	0.9566	0.8968	0.7587	0.6739	0.6934	0.4513	0.9556	0.8969
SIDM00638	0.9516	0.8349	0.9516	0.8349	0.7974	0.6688	0.6888	0.4578	0.9477	0.8199
SIDM00579	0.9082	0.888	0.9082	0.888	0.666	0.66	0.6902	0.4517	0.9092	0.8887
SIDM00762	0.9533	0.876	0.9533	0.876	0.8239	0.7653	0.6943	0.4548	0.9576	0.8912
SIDM00761	0.8964	0.5757	0.8964	0.5757	0.6161	0.3892	0.691	0.4478	0.9018	0.5917
SIDM00753	0.9292	0.8569	0.9292	0.8569	0.7321	0.6752	0.7009	0.4504	0.9191	0.8405
SIDM00704	0.8447	0.8531	0.8447	0.8531	0.5748	0.6329	0.6974	0.4533	0.8324	0.8471
SIDM00137	0.942	0.8836	0.942	0.8836	0.647	0.5252	0.7025	0.4489	0.941	0.8847
SIDM01149	0.9629	0.9669	0.9629	0.9669	0.7429	0.7105	0.7082	0.4628	0.96	0.9675
SIDM00565	0.9464	0.8576	0.9464	0.8576	0.7527	0.5389	0.7079	0.4659	0.9478	0.8711
SIDM01141	0.8287	0.6403	0.8287	0.6403	0.7809	0.5173	0.7092	0.4572	0.8086	0.6269
SIDM00564	0.9438	0.8586	0.9438	0.8586	0.7124	0.461	0.6911	0.4493	0.9438	0.8673
SIDM00466	0.9815	0.75	0.9815	0.75	0.5833	0.0625	0.7084	0.4634	0.9815	0.75
SIDM00465	0.8493	0.7768	0.8493	0.7768	0.731	0.6542	0.6986	0.4507	0.8423	0.7667
SIDM00453	0.9601	0.933	0.9601	0.933	0.9117	0.904	0.6915	0.4522	0.9601	0.9361
SIDM00358	0.8847	0.8604	0.8847	0.8604	0.6825	0.522	0.6912	0.4553	0.8898	0.8691

Continued on next page

Table S8 – continued from previous page

Cell line	Ridge		Lasso		CNN		tCNN		BKDRP	
	AUC	AUPR	AUC	AUPR	AUC	AUPR	AUC	AUPR	AUC	AUPR
SIDM00394	0.9505	0.8547	0.9505	0.8547	0.7967	0.5969	0.7079	0.4709	0.9464	0.8405
SIDM01181	0.8706	0.7992	0.8706	0.7992	0.7149	0.6047	0.7055	0.4665	0.8635	0.7826
SIDM01188	0.7218	0.3353	0.7218	0.3353	0.5484	0.0941	0.7174	0.467	0.7137	0.3331
SIDM00371	0.8791	0.6679	0.8791	0.6679	0.7446	0.363	0.6995	0.4579	0.8733	0.6398
SIDM01169	0.8848	0.8402	0.8848	0.8402	0.6882	0.5874	0.7013	0.4638	0.87	0.8299
SIDM00418	0.8039	0.4328	0.8039	0.4328	0.7651	0.2874	0.7064	0.4587	0.8039	0.4422
SIDM00975	0.8715	0.7753	0.8715	0.7753	0.7347	0.5995	0.6975	0.4566	0.8655	0.7698
SIDM01176	0.8116	0.8339	0.8116	0.8339	0.7869	0.7811	0.6924	0.4473	0.8068	0.8246
SIDM00491	0.9608	0.9155	0.9608	0.9155	0.8891	0.8132	0.702	0.46	0.9597	0.9143
SIDM00936	0.9727	0.9709	0.9727	0.9709	0.782	0.7642	0.7071	0.4611	0.9718	0.9705
SIDM00944	0.8993	0.8067	0.8993	0.8067	0.7216	0.4736	0.6975	0.4558	0.8915	0.7995
SIDM00950	0.9573	0.92	0.9573	0.92	0.7358	0.6843	0.6948	0.4479	0.9644	0.9308
SIDM01002	0.8345	0.7181	0.8333	0.7162	0.7789	0.5433	0.6908	0.448	0.8356	0.7137
SIDM00503	0.8594	0.7949	0.8594	0.7949	0.5953	0.3461	0.7084	0.4599	0.8578	0.792
SIDM00104	0.9322	0.8351	0.9322	0.8351	0.7603	0.5934	0.6965	0.453	0.9306	0.8219
SIDM00103	0.963	0.9353	0.9619	0.933	0.837	0.7786	0.6933	0.4481	0.9598	0.9283
SIDM00102	0.9176	0.737	0.9176	0.737	0.7651	0.6105	0.7097	0.4643	0.9245	0.7578
SIDM00099	0.95	0.6885	0.95	0.6885	0.7111	0.1821	0.7031	0.5174	0.9639	0.7404
SIDM00154	0.9449	0.9505	0.9449	0.9505	0.7289	0.8018	0.7092	0.5216	0.9485	0.953
SIDM00520	0.9472	0.8091	0.9472	0.8091	0.6831	0.4924	0.7084	0.5156	0.952	0.8179
SIDM00881	0.8095	0.4737	0.8095	0.4737	0.3405	0.0856	0.7222	0.5274	0.8214	0.5119
SIDM01068	0.9058	0.8739	0.9058	0.8739	0.6964	0.5791	0.7095	0.521	0.9058	0.8741
SIDM01062	0.859	0.8452	0.859	0.8452	0.7143	0.5861	0.711	0.5253	0.8471	0.8284
SIDM00994	0.9051	0.9289	0.9051	0.9289	0.7011	0.7366	0.7097	0.5218	0.9098	0.9343
SIDM00993	0.9395	0.9641	0.9395	0.9641	0.6759	0.7117	0.719	0.5239	0.9303	0.9606
SIDM00946	0.8472	0.8438	0.8462	0.8434	0.6179	0.5493	0.7057	0.5226	0.8416	0.8358
SIDM01048	0.9504	0.9307	0.9504	0.9307	0.749	0.6117	0.7152	0.5249	0.9535	0.9363
SIDM00846	0.91	0.7733	0.91	0.7733	0.6851	0.4474	0.7039	0.5196	0.91	0.7631
SIDM00670	0.9046	0.7747	0.9046	0.7747	0.783	0.483	0.7077	0.5121	0.898	0.7574
SIDM00664	0.9005	0.7344	0.9005	0.7344	0.6088	0.2603	0.7137	0.5254	0.8912	0.7201
SIDM00586	0.8592	0.7302	0.8592	0.7302	0.7242	0.4658	0.6981	0.5227	0.8476	0.7238
SIDM00618	0.939	0.7846	0.939	0.7846	0.7997	0.4767	0.7179	0.5284	0.9376	0.7803
SIDM01037	0.9323	0.8656	0.9323	0.8656	0.5172	0.4045	0.7212	0.5305	0.9364	0.8717
SIDM00611	0.8927	0.7503	0.8927	0.7503	0.4508	0.2281	0.6859	0.5047	0.898	0.7565
SIDM00609	0.9543	0.8802	0.9543	0.8802	0.8522	0.7303	0.7202	0.5297	0.9478	0.8604
SIDM00583	0.9507	0.8802	0.9507	0.8802	0.8099	0.5341	0.7106	0.5242	0.9419	0.8576
SIDM00582	0.9036	0.7727	0.9036	0.7727	0.7464	0.4479	0.7072	0.5239	0.8929	0.7636
SIDM00550	0.9261	0.8543	0.9261	0.8543	0.7522	0.6248	0.721	0.5309	0.9304	0.8549
SIDM00341	0.9337	0.926	0.9337	0.926	0.7454	0.7178	0.7116	0.5236	0.9376	0.9292
SIDM00658	0.9419	0.6136	0.9419	0.6136	0.7191	0.287	0.7146	0.5236	0.9443	0.62
SIDM00750	0.8519	0.6779	0.8519	0.6779	0.7315	0.4775	0.7305	0.5443	0.8488	0.6748
SIDM00792	0.896	0.6343	0.896	0.6343	0.4113	0.149	0.6985	0.5116	0.8936	0.6319
SIDM00754	0.8243	0.7514	0.8243	0.7514	0.764	0.5636	0.7113	0.5125	0.8233	0.7542
SIDM00570	0.8161	0.4447	0.8161	0.4447	0.6518	0.3839	0.7192	0.521	0.8036	0.4341
SIDM00568	0.9124	0.7994	0.9124	0.7994	0.7752	0.6156	0.7013	0.5215	0.915	0.8043
SIDM00478	0.8876	0.8877	0.8876	0.8877	0.7382	0.7737	0.7213	0.53	0.8903	0.8851
SIDM00425	0.9475	0.8772	0.9475	0.8772	0.6825	0.477	0.6929	0.5117	0.9475	0.8716
SIDM01139	0.9009	0.8539	0.9009	0.8539	0.7583	0.6894	0.7249	0.536	0.9009	0.8522
SIDM01137	0.8105	0.5128	0.8105	0.5128	0.5686	0.1552	0.7111	0.5233	0.8137	0.5144
SIDM00455	0.9657	0.9176	0.9657	0.9176	0.794	0.5674	0.703	0.521	0.9739	0.935
SIDM00044	0.9565	0.8227	0.9565	0.8227	0.9196	0.8275	0.718	0.524	0.9522	0.8109
SIDM00042	0.9514	0.9195	0.9514	0.9195	0.7698	0.7031	0.7123	0.5219	0.9593	0.9334
SIDM00356	0.9201	0.8039	0.9201	0.8039	0.8079	0.6947	0.6909	0.5056	0.9132	0.798
SIDM00354	0.8322	0.8	0.8322	0.8	0.7008	0.6641	0.7195	0.5285	0.8322	0.7998
SIDM00350	0.8233	0.7343	0.8233	0.7343	0.6798	0.5908	0.7145	0.516	0.8182	0.7296

Continued on next page

Table S8 – continued from previous page

Cell line	Ridge		Lasso		CNN		tCNN		BKDRP	
	AUC	AUPR	AUC	AUPR	AUC	AUPR	AUC	AUPR	AUC	AUPR
SIDM00399	0.7541	0.4638	0.7541	0.4638	0.7157	0.4966	0.7191	0.522	0.7473	0.461
SIDM00375	0.976	0.9529	0.976	0.9529	0.5275	0.3496	0.7145	0.5274	0.976	0.9486
SIDM01156	0.9162	0.7429	0.9162	0.7429	0.7329	0.499	0.7055	0.5185	0.9201	0.7667
SIDM00322	0.8949	0.8776	0.8949	0.8776	0.6282	0.57	0.7068	0.5231	0.8923	0.8721
SIDM01170	0.9317	0.8806	0.9317	0.8806	0.7317	0.6733	0.6997	0.5141	0.9239	0.869
SIDM01077	0.9163	0.8303	0.915	0.8277	0.795	0.5903	0.7144	0.5235	0.9013	0.8083
SIDM00976	0.9731	0.9434	0.9731	0.9434	0.9068	0.8872	0.7217	0.5314	0.9741	0.9449
SIDM00974	0.9116	0.8766	0.9116	0.8766	0.6011	0.5035	0.72	0.5252	0.9053	0.8692
SIDM00973	0.9786	0.9766	0.9786	0.9766	0.7782	0.7953	0.7229	0.5343	0.9758	0.9727
SIDM01058	0.8199	0.6185	0.8199	0.6185	0.5858	0.3419	0.7101	0.5227	0.8235	0.605
SIDM00879	0.8665	0.6358	0.865	0.6101	0.7054	0.4497	0.7184	0.5276	0.8766	0.6306
SIDM00508	0.9049	0.905	0.9049	0.905	0.6627	0.5895	0.7169	0.5219	0.9169	0.9146
SIDM00045	0.9538	0.9731	0.9538	0.9731	0.6106	0.6586	0.717	0.534	0.9496	0.9708
SIDM01036	0.9671	0.9601	0.9671	0.9601	0.8186	0.8143	0.7044	0.5109	0.9643	0.9567
SIDM00688	0.9195	0.9322	0.9195	0.9322	0.6042	0.6144	0.6999	0.5149	0.9091	0.9196
SIDM01011	0.9091	0.9221	0.9091	0.9221	0.6713	0.6852	0.7006	0.5126	0.9073	0.9214
SIDM01006	0.8824	0.8707	0.8824	0.8707	0.7105	0.7258	0.7141	0.5168	0.8888	0.8718
SIDM01004	0.9375	0.9423	0.9375	0.9423	0.6691	0.7387	0.7098	0.5271	0.9311	0.9381
SIDM00338	0.7731	0.4788	0.7731	0.4788	0.5818	0.2988	0.7107	0.522	0.7639	0.4554
SIDM00437	0.9121	0.9754	0.9121	0.9754	0.6236	0.8739	0.7224	0.523	0.9245	0.9792
SIDM00434	0.9454	0.9589	0.9454	0.9589	0.737	0.8004	0.7196	0.5207	0.9426	0.9574
SIDM00646	0.8805	0.8023	0.8805	0.8023	0.7368	0.5519	0.7141	0.5161	0.8677	0.7795
SIDM00711	0.9213	0.7544	0.9213	0.7544	0.481	0.1353	0.7177	0.5211	0.9329	0.7847
SIDM00458	0.9087	0.9327	0.9087	0.9327	0.7614	0.841	0.7155	0.5186	0.9068	0.928
SIDM00374	0.9032	0.9154	0.9032	0.9154	0.6737	0.701	0.7051	0.5147	0.9014	0.9137
SIDM00389	0.9609	0.9708	0.9609	0.9708	0.7493	0.8166	0.6943	0.5054	0.9609	0.972
SIDM00407	0.8995	0.9337	0.8995	0.9337	0.6072	0.6803	0.6978	0.5183	0.9077	0.9419
SIDM00423	0.8532	0.8022	0.8532	0.8022	0.6062	0.5083	0.7149	0.5225	0.8442	0.7955
SIDM00323	0.8815	0.8005	0.8815	0.8005	0.6169	0.5273	0.7042	0.5175	0.8889	0.804
SIDM00416	0.8454	0.8645	0.8454	0.8645	0.6139	0.6516	0.7114	0.5179	0.8537	0.8683
SIDM00413	0.914	0.943	0.914	0.943	0.6277	0.709	0.7064	0.5186	0.9207	0.9476
SIDM00969	0.9469	0.9422	0.9469	0.9422	0.7036	0.6877	0.7035	0.5221	0.9432	0.9428
SIDM00968	0.9483	0.9203	0.9483	0.9203	0.7473	0.754	0.7149	0.5267	0.9493	0.9257
SIDM00475	0.9104	0.7204	0.9104	0.7204	0.8596	0.6717	0.7015	0.5144	0.9128	0.7282
SIDM00967	0.7978	0.7336	0.7978	0.7336	0.462	0.3962	0.721	0.5263	0.7913	0.7234
SIDM00472	0.9202	0.7539	0.9202	0.7539	0.836	0.5822	0.7012	0.5154	0.9274	0.7639
SIDM01041	0.9452	0.923	0.9452	0.923	0.7645	0.6816	0.7188	0.5237	0.9432	0.9205
SIDM00445	0.9513	0.9534	0.9513	0.9534	0.8456	0.8628	0.717	0.5324	0.9513	0.9523
SIDM00444	0.8741	0.8532	0.8741	0.8532	0.8093	0.7421	0.7355	0.5385	0.8731	0.8541
SIDM00393	0.8915	0.8974	0.8915	0.8974	0.7445	0.7766	0.7161	0.5254	0.8925	0.8973
SIDM00483	0.8941	0.7313	0.8929	0.7291	0.6008	0.3391	0.7191	0.5317	0.9005	0.7469
SIDM00539	0.9304	0.7943	0.9304	0.7943	0.7803	0.6617	0.7138	0.517	0.9268	0.8007
SIDM00538	0.9058	0.8817	0.9058	0.8817	0.7381	0.715	0.6987	0.4922	0.8948	0.8664
SIDM00530	0.8067	0.6037	0.8067	0.6037	0.6505	0.3976	0.6868	0.4775	0.8113	0.613
SIDM00047	0.9519	0.9355	0.9519	0.9355	0.7712	0.7348	0.6994	0.4877	0.9471	0.9318
SIDM00250	0.9096	0.8713	0.9096	0.8713	0.7327	0.6961	0.6904	0.4809	0.9115	0.8673
SIDM00817	0.9616	0.9511	0.9616	0.9511	0.73	0.6704	0.7003	0.4903	0.9616	0.9487
SIDM01265	0.9207	0.9448	0.9217	0.945	0.7032	0.7422	0.7048	0.4923	0.9226	0.9466
SIDM00216	0.9484	0.9496	0.9484	0.9496	0.7696	0.7521	0.6943	0.4877	0.9465	0.9483
SIDM00214	0.9199	0.8401	0.9199	0.8401	0.5325	0.3748	0.7053	0.4905	0.9113	0.8268
SIDM00194	0.8765	0.6628	0.8765	0.6628	0.4784	0.1691	0.6836	0.4879	0.8667	0.6632
SIDM00498	0.9831	0.9709	0.9831	0.9709	0.8423	0.7895	0.6933	0.4827	0.9802	0.9683
SIDM00049	0.8553	0.8316	0.8553	0.8316	0.7951	0.7741	0.694	0.4883	0.8543	0.8251
SIDM00807	0.9438	0.9305	0.9428	0.9282	0.5611	0.5612	0.6915	0.486	0.9438	0.9258
SIDM00315	0.9167	0.814	0.9167	0.814	0.7333	0.517	0.6922	0.487	0.9222	0.8235

Continued on next page

Table S8 – continued from previous page

Cell line	Ridge		Lasso		CNN		tCNN		BKDRP	
	AUC	AUPR	AUC	AUPR	AUC	AUPR	AUC	AUPR	AUC	AUPR
SIDM00734	0.8947	0.8653	0.8947	0.8653	0.7842	0.7912	0.6928	0.485	0.8937	0.8668
SIDM00735	0.8994	0.74	0.8994	0.74	0.6445	0.3836	0.6887	0.4799	0.8945	0.732
SIDM00733	0.9313	0.9353	0.9313	0.9353	0.6698	0.6777	0.6886	0.481	0.924	0.9321
SIDM00730	0.8532	0.6781	0.8532	0.6781	0.5532	0.3992	0.6909	0.4833	0.8564	0.6843
SIDM00719	0.8889	0.4361	0.8889	0.4361	0.3056	0.0654	0.6845	0.4739	0.8929	0.4394
SIDM00293	0.9167	0.8424	0.9167	0.8424	0.5854	0.4045	0.7013	0.4905	0.9208	0.8555
SIDM00365	0.9135	0.8757	0.9135	0.8757	0.6314	0.5151	0.7004	0.4912	0.9092	0.8732
SIDM00715	0.8659	0.7938	0.8659	0.7938	0.8038	0.7615	0.7048	0.4989	0.8758	0.8079
SIDM00739	0.9178	0.8862	0.9178	0.8862	0.7395	0.699	0.6977	0.4861	0.9222	0.8963
SIDM01184	0.9298	0.7052	0.9298	0.7052	0.8743	0.4481	0.6925	0.4814	0.9357	0.687
SIDM00728	0.8114	0.4384	0.8114	0.4384	0.6545	0.2051	0.6915	0.4783	0.8227	0.4683
SIDM00877	0.8888	0.8312	0.8888	0.8312	0.823	0.6912	0.7028	0.4942	0.8816	0.8297
SIDM00772	0.809	0.605	0.809	0.605	0.7522	0.5579	0.6897	0.4833	0.8149	0.6082
SIDM00172	0.9326	0.956	0.9326	0.956	0.7032	0.7971	0.6997	0.4887	0.9379	0.9598
SIDM00191	0.9213	0.8196	0.9213	0.8196	0.8644	0.7047	0.6992	0.4882	0.9242	0.8231
SIDM00986	0.8868	0.7116	0.8868	0.7116	0.4472	0.2371	0.6933	0.4815	0.8849	0.7107
SIDM00175	0.9168	0.9094	0.9168	0.9094	0.6724	0.5965	0.6996	0.4849	0.9138	0.9172
SIDM00190	0.8649	0.6228	0.8649	0.6228	0.5456	0.1767	0.7021	0.4937	0.8649	0.6081
SIDM00207	0.9258	0.8359	0.9258	0.8359	0.5627	0.3686	0.6999	0.4841	0.922	0.8356
SIDM00524	0.7735	0.494	0.7735	0.494	0.3704	0.1853	0.7092	0.4938	0.7792	0.524
SIDM00188	0.9265	0.7424	0.9265	0.7424	0.8444	0.5437	0.6901	0.4943	0.9265	0.7653
SIDM00052	0.9543	0.8992	0.9543	0.8992	0.6062	0.3969	0.7082	0.5077	0.9568	0.9041
SIDM00223	0.8977	0.8326	0.8977	0.8326	0.567	0.397	0.6931	0.4758	0.8807	0.7988
SIDM00603	0.9485	0.9503	0.9485	0.9503	0.5889	0.5545	0.6865	0.4823	0.9515	0.9523
SIDM00187	0.8624	0.7773	0.8624	0.7773	0.4952	0.3297	0.6972	0.4897	0.8696	0.7841
SIDM00186	0.9417	0.8725	0.9417	0.8725	0.9264	0.8416	0.7049	0.4913	0.9389	0.8671
SIDM00185	0.8834	0.7846	0.8834	0.7846	0.7609	0.6208	0.695	0.4904	0.8776	0.7742
SIDM00184	0.8597	0.5718	0.8597	0.5718	0.5051	0.2619	0.6996	0.4869	0.852	0.5662
SIDM00183	0.9199	0.8109	0.9199	0.8109	0.5239	0.3522	0.6923	0.4878	0.9115	0.7915
SIDM00296	0.9082	0.9113	0.9082	0.9113	0.5367	0.4896	0.6905	0.4754	0.9071	0.9091
SIDM00053	0.959	0.6429	0.959	0.6429	0.3361	0.0352	0.7031	0.4915	0.959	0.6429
SIDM00516	0.817	0.749	0.817	0.749	0.4958	0.4242	0.6974	0.4901	0.8181	0.7524
SIDM00927	0.8432	0.687	0.8432	0.687	0.537	0.3532	0.7	0.4878	0.8346	0.682
SIDM00249	0.9342	0.9313	0.9342	0.9313	0.7011	0.6083	0.688	0.4824	0.9342	0.932
SIDM00349	0.9104	0.8781	0.9104	0.8781	0.6327	0.476	0.7017	0.4958	0.9104	0.8907
SIDM00081	0.8888	0.8609	0.8888	0.8609	0.7931	0.8197	0.7088	0.4886	0.8898	0.8667
SIDM00080	0.9058	0.8243	0.9058	0.8243	0.5704	0.3841	0.703	0.4914	0.9037	0.8223
SIDM00941	0.8481	0.7992	0.8481	0.7992	0.5079	0.3701	0.6981	0.4904	0.8503	0.7994
SIDM00119	0.75	0.3859	0.75	0.3859	0.6	0.1599	0.6903	0.4811	0.75	0.4308
SIDM00086	0.9153	0.7564	0.9153	0.7564	0.6424	0.4037	0.7022	0.4897	0.9153	0.7584
SIDM00112	0.8631	0.8546	0.8631	0.8546	0.4604	0.4376	0.6943	0.4917	0.858	0.8517
SIDM00095	0.8896	0.7865	0.8896	0.7865	0.625	0.3329	0.704	0.498	0.8883	0.7904
SIDM00084	0.8939	0.7998	0.8939	0.7998	0.5946	0.4781	0.6874	0.4859	0.89	0.7909
SIDM00494	0.9387	0.7131	0.9387	0.7131	0.5019	0.279	0.6986	0.493	0.9464	0.7582
SIDM00952	0.8747	0.9168	0.8747	0.9168	0.6122	0.661	0.6888	0.4811	0.8834	0.9243
SIDM00515	0.8716	0.7846	0.8716	0.7846	0.8123	0.624	0.6862	0.4806	0.8741	0.7907
SIDM00919	0.8947	0.7828	0.8947	0.7828	0.6208	0.3995	0.6942	0.4888	0.9067	0.8076
SIDM00981	0.8915	0.9378	0.8924	0.9381	0.6172	0.7747	0.6898	0.4824	0.8963	0.9405
SIDM00878	0.9238	0.8855	0.9238	0.8855	0.599	0.485	0.6883	0.4855	0.9152	0.8649
SIDM00594	0.8295	0.7503	0.8295	0.7503	0.3905	0.3189	0.6863	0.4816	0.8324	0.7557
SIDM00661	0.8736	0.8841	0.8736	0.8841	0.534	0.5056	0.6969	0.4855	0.8736	0.8855
SIDM01059	0.8921	0.8553	0.8921	0.8553	0.8632	0.8447	0.7046	0.4928	0.8814	0.8454
SIDM01005	0.8616	0.9363	0.8636	0.9369	0.6545	0.7511	0.7049	0.4915	0.8374	0.9222
SIDM00607	0.8954	0.6019	0.8954	0.6019	0.6454	0.2655	0.6852	0.4835	0.8954	0.6106
SIDM00534	0.8872	0.8039	0.8872	0.8039	0.567	0.3444	0.687	0.4759	0.8745	0.7913

Continued on next page

Cell line	Ridge		Lasso		CNN		tCNN		BKDRP	
	AUC	AUPR	AUC	AUPR	AUC	AUPR	AUC	AUPR	AUC	AUPR
SIDM01026	0.9529	0.8339	0.9529	0.8339	0.6641	0.5183	0.6897	0.4816	0.9582	0.8454
SIDM00297	0.9391	0.9075	0.9391	0.9075	0.4893	0.4755	0.6946	0.4838	0.9402	0.9148
SIDM00340	0.9088	0.7762	0.9088	0.7762	0.6994	0.5167	0.7465	0.5203	0.9031	0.7705
SIDM00677	0.93	0.8974	0.93	0.8974	0.652	0.5816	0.7557	0.5385	0.924	0.893
SIDM00637	0.9212	0.9369	0.9212	0.9369	0.6019	0.6444	0.7332	0.522	0.9191	0.9361
SIDM01246	0.8697	0.864	0.8697	0.864	0.6616	0.6156	0.7602	0.5259	0.8737	0.868
SIDM00337	0.8492	0.7653	0.8492	0.7653	0.643	0.5246	0.7602	0.5259	0.837	0.7495
SIDM00777	0.8515	0.8509	0.8505	0.8506	0.8515	0.8266	0.7293	0.509	0.8485	0.8526
SIDM00575	0.85	0.4686	0.85	0.4686	0.2667	0.042	0.7133	0.5002	0.8444	0.467
SIDM00573	0.8793	0.7104	0.8793	0.7104	0.6517	0.3507	0.7354	0.5111	0.8862	0.7378
SIDM00574	0.8259	0.6291	0.8259	0.6291	0.579	0.4028	0.7537	0.5408	0.8259	0.6348
SIDM00652	0.8235	0.5699	0.8235	0.5699	0.6993	0.3855	0.753	0.5355	0.8121	0.5265
SIDM00697	0.8876	0.7409	0.8876	0.7409	0.5239	0.3697	0.6935	0.5392	0.8816	0.7382
SIDM00710	0.8889	0.8095	0.8889	0.8095	0.644	0.4825	0.7001	0.5414	0.9025	0.8349
SIDM01117	0.8299	0.7461	0.8299	0.7461	0.5	0.2916	0.6843	0.5276	0.8338	0.748
SIDM00401	0.8418	0.5246	0.8418	0.5246	0.5638	0.204	0.6924	0.5327	0.8418	0.5236
SIDM00382	0.9168	0.8821	0.9168	0.8821	0.6143	0.5701	0.7039	0.5416	0.9189	0.8896
SIDM00396	0.9021	0.9155	0.9021	0.9155	0.5773	0.6045	0.6908	0.5362	0.901	0.9169
SIDM01105	0.8333	0.7661	0.8333	0.7661	0.5844	0.4713	0.6858	0.528	0.8205	0.7593
SIDM00392	0.836	0.6981	0.836	0.6981	0.6291	0.4551	0.6949	0.5375	0.8337	0.6966
SIDM00370	0.9023	0.9579	0.9023	0.9579	0.6267	0.7414	0.6971	0.5324	0.8953	0.9546
SIDM01172	0.9105	0.8942	0.9105	0.8942	0.5525	0.4892	0.7151	0.5492	0.9105	0.8905
SIDM01186	0.7579	0.6466	0.7579	0.6466	0.6168	0.5313	0.6987	0.5352	0.7589	0.6551
SIDM01150	0.929	0.9037	0.929	0.9037	0.57	0.5231	0.6922	0.5323	0.9259	0.9041
SIDM00482	0.9314	0.9462	0.9314	0.9462	0.6381	0.6793	0.7038	0.5461	0.9295	0.9447
SIDM00286	0.8036	0.658	0.8036	0.658	0.5274	0.3327	0.6891	0.5346	0.8131	0.6882
SIDM01082	0.8444	0.5456	0.8444	0.5456	0.5357	0.206	0.7087	0.537	0.8469	0.5465
SIDM01089	0.9048	0.9228	0.9048	0.9228	0.6948	0.7683	0.6913	0.5278	0.9102	0.9283
SIDM00698	0.8983	0.8433	0.8983	0.8433	0.5514	0.4063	0.6968	0.5414	0.9007	0.8391
SIDM00895	0.9047	0.8928	0.9047	0.8928	0.5477	0.5345	0.6913	0.5299	0.9057	0.8982
SIDM00355	0.9202	0.9225	0.9202	0.9225	0.6697	0.6416	0.6841	0.525	0.9061	0.9141
SIDM01210	0.9643	0.8075	0.9643	0.8075	0.8469	0.5825	0.6896	0.5297	0.9694	0.8469
SIDM00557	0.7991	0.7211	0.7991	0.7211	0.5791	0.5264	0.6972	0.5351	0.8002	0.7258
SIDM00721	0.9298	0.8007	0.9298	0.8007	0.6009	0.4163	0.686	0.5277	0.9276	0.8038
SIDM01242	0.8061	0.5946	0.8061	0.5946	0.6913	0.2969	0.6824	0.5249	0.801	0.5753
SIDM00319	0.8989	0.8328	0.8989	0.8328	0.4375	0.2337	0.6993	0.5408	0.891	0.8244
SIDM00348	0.9343	0.9362	0.9343	0.9362	0.5616	0.5655	0.7037	0.533	0.9323	0.9333
SIDM00023	0.8525	0.755	0.8525	0.755	0.6242	0.4782	0.6871	0.5269	0.8545	0.7582
SIDM00157	0.8306	0.8032	0.8306	0.8032	0.5395	0.5092	0.6856	0.5307	0.8274	0.7944
SIDM00622	0.907	0.849	0.907	0.849	0.7891	0.7196	0.699	0.5374	0.9104	0.8568
SIDM00620	0.8519	0.8035	0.8519	0.8035	0.6914	0.589	0.6845	0.5269	0.8488	0.8007
SIDM00209	0.9023	0.8691	0.9023	0.8691	0.5432	0.4778	0.6892	0.535	0.9095	0.8769
SIDM00208	0.8994	0.7588	0.8994	0.7588	0.7813	0.5446	0.7004	0.5422	0.9009	0.7733
SIDM00206	0.9163	0.8729	0.9163	0.8729	0.6098	0.4824	0.6917	0.5356	0.9196	0.8801
SIDM00283	0.8449	0.8643	0.8449	0.8643	0.6622	0.7093	0.6982	0.5324	0.85	0.8654
SIDM00240	0.9017	0.87	0.9017	0.87	0.5513	0.4153	0.6786	0.5287	0.9103	0.8848
SIDM00330	0.8116	0.6714	0.8116	0.6714	0.5267	0.4033	0.7107	0.5479	0.8081	0.6687
SIDM00253	0.7658	0.7526	0.7668	0.7529	0.6397	0.5911	0.6912	0.5331	0.7668	0.7557
SIDM00255	0.8486	0.8254	0.8486	0.8254	0.5512	0.4865	0.7206	0.5554	0.8497	0.8267
SIDM00254	0.9184	0.8414	0.9184	0.8414	0.5816	0.4144	0.68	0.5251	0.9206	0.8502
SIDM00179	0.8818	0.8781	0.8818	0.8781	0.6657	0.6481	0.7048	0.5419	0.8798	0.8806
SIDM00955	0.8776	0.7973	0.8776	0.7973	0.6304	0.4215	0.6877	0.5292	0.858	0.7733
SIDM00129	0.9167	0.8991	0.9167	0.8991	0.6685	0.626	0.7025	0.5343	0.9149	0.9008
SIDM00309	0.947	0.8498	0.947	0.8498	0.6955	0.3301	0.6903	0.5299	0.9515	0.8574
SIDM00310	0.9439	0.8538	0.9439	0.8538	0.5136	0.2615	0.6844	0.52	0.9409	0.8363

Continued on next page

Table S8 – continued from previous page

Cell line	Ridge		Lasso		CNN		tCNN		BKDRP	
	AUC	AUPR	AUC	AUPR	AUC	AUPR	AUC	AUPR	AUC	AUPR
SIDM00311	0.8969	0.7113	0.8969	0.7113	0.5636	0.4171	0.7019	0.5433	0.8925	0.7008
SIDM00519	0.9439	0.8098	0.9439	0.8098	0.5596	0.1816	0.6907	0.5276	0.9474	0.8056
SIDM00518	0.9255	0.8311	0.9255	0.8311	0.5678	0.3268	0.6779	0.5235	0.9215	0.8246
SIDM01072	0.8725	0.685	0.8725	0.685	0.6422	0.3889	0.6752	0.5225	0.8676	0.6796
SIDM00672	0.9064	0.8963	0.9064	0.8963	0.6488	0.5867	0.6896	0.525	0.9055	0.8988
SIDM00666	0.8916	0.8411	0.8916	0.8411	0.7495	0.7158	0.7114	0.5467	0.8947	0.8493
SIDM00128	0.9259	0.9445	0.9259	0.9445	0.6811	0.7089	0.6896	0.5364	0.9239	0.9443
SIDM01220	0.9167	0.8448	0.9167	0.8448	0.6754	0.6241	0.6882	0.5626	0.8991	0.8259
SIDM00442	0.8698	0.6415	0.8698	0.6415	0.4792	0.2043	0.6965	0.5699	0.8679	0.6407
SIDM00770	0.928	0.6563	0.928	0.6563	0.7203	0.2303	0.6952	0.5692	0.9322	0.6604
SIDM00270	0.9356	0.9548	0.9356	0.9548	0.5894	0.6493	0.706	0.5776	0.9356	0.9543
SIDM00115	0.8837	0.8277	0.8837	0.8277	0.5844	0.5016	0.6903	0.5629	0.8827	0.8216
SIDM01162	0.8776	0.6254	0.8776	0.6254	0.4615	0.1964	0.687	0.5656	0.8794	0.6246
SIDM00318	0.8655	0.6583	0.8655	0.6583	0.6228	0.3397	0.6909	0.557	0.8626	0.6361
SIDM00046	0.7397	0.4961	0.7397	0.4961	0.5962	0.34	0.6967	0.5741	0.7321	0.4844
SIDM01240	0.8265	0.6165	0.8265	0.6165	0.7187	0.4562	0.7027	0.5735	0.828	0.6125
SIDM00078	0.8777	0.6634	0.8777	0.6634	0.5013	0.2602	0.6861	0.561	0.8737	0.6554
SIDM00510	0.9262	0.9269	0.9262	0.9269	0.6736	0.5978	0.6808	0.5619	0.9231	0.9257
SIDM00521	0.8067	0.6612	0.8067	0.6612	0.4333	0.1927	0.7023	0.5694	0.8	0.6296
SIDM00859	0.9122	0.9429	0.9122	0.9429	0.6966	0.8039	0.6901	0.571	0.9132	0.944
SIDM00663	0.8868	0.8738	0.8868	0.8738	0.8035	0.7973	0.6873	0.5641	0.8889	0.8729
SIDM00662	0.8617	0.772	0.8617	0.772	0.5284	0.3601	0.7022	0.5766	0.8654	0.7787
SIDM00690	0.923	0.9321	0.923	0.9321	0.5567	0.5608	0.6975	0.5653	0.9147	0.9274
SIDM00126	0.8359	0.7729	0.8359	0.7729	0.6563	0.506	0.6982	0.5768	0.8381	0.7808
SIDM00619	0.9253	0.8675	0.9253	0.8675	0.5989	0.4719	0.6838	0.5563	0.9263	0.8719
SIDM00604	0.9204	0.9147	0.9204	0.9147	0.6139	0.5885	0.6993	0.5746	0.9133	0.9084
SIDM00566	0.9337	0.9057	0.9337	0.9057	0.5717	0.438	0.7079	0.5767	0.9261	0.8956
SIDM00043	0.9037	0.8657	0.9028	0.8637	0.5185	0.4351	0.6956	0.5674	0.9102	0.8765
SIDM00040	0.9291	0.8719	0.9291	0.8719	0.5698	0.3789	0.7056	0.5808	0.9326	0.8799
SIDM01071	0.9246	0.8247	0.9246	0.8247	0.5537	0.3062	0.7041	0.5791	0.9233	0.8208
SIDM00428	0.857	0.9337	0.8593	0.9343	0.6116	0.7462	0.7021	0.5795	0.8651	0.9381
SIDM01038	0.873	0.8917	0.875	0.8924	0.5252	0.5033	0.704	0.5743	0.877	0.8992
SIDM00689	0.8802	0.8146	0.8802	0.8146	0.5105	0.4419	0.6863	0.5624	0.8733	0.8118
SIDM01015	0.9294	0.951	0.9294	0.951	0.5529	0.7252	0.702	0.5702	0.9314	0.9487
SIDM00631	0.8635	0.7893	0.8635	0.7893	0.7037	0.6364	0.6835	0.5568	0.8615	0.7942
SIDM00435	0.8555	0.9007	0.8555	0.9007	0.5572	0.6183	0.6988	0.5738	0.8669	0.9096
SIDM01217	0.8083	0.8221	0.8083	0.8221	0.6865	0.713	0.6907	0.5669	0.8167	0.8323
SIDM00443	0.8515	0.8976	0.8515	0.8976	0.5556	0.6251	0.695	0.5696	0.8611	0.9082
SIDM00461	0.9269	0.9486	0.9269	0.9486	0.5788	0.6354	0.7048	0.5715	0.9231	0.9464
SIDM00460	0.8273	0.8337	0.8273	0.8337	0.5212	0.5261	0.6984	0.5707	0.8141	0.8228
SIDM00450	0.9306	0.9342	0.9306	0.9342	0.6755	0.7036	0.7062	0.5776	0.9357	0.9441
SIDM00404	0.9174	0.945	0.9174	0.945	0.6185	0.7343	0.7052	0.5707	0.9207	0.9474
SIDM00405	0.9185	0.9358	0.9185	0.9358	0.5947	0.6607	0.6976	0.573	0.9249	0.9418
SIDM00305	0.9477	0.9248	0.9477	0.9248	0.6086	0.5343	0.7006	0.5706	0.9524	0.9355
SIDM00304	0.8654	0.6559	0.8654	0.6559	0.5122	0.2049	0.701	0.5772	0.8654	0.6639
SIDM00303	0.9222	0.9347	0.9222	0.9347	0.5505	0.5592	0.698	0.5707	0.9242	0.9381
SIDM00474	0.9274	0.8742	0.9274	0.8742	0.5952	0.428	0.7023	0.5744	0.9331	0.884
SIDM00476	0.9207	0.8504	0.9207	0.8504	0.5946	0.3493	0.709	0.5788	0.9233	0.8557
SIDM00966	0.947	0.9212	0.947	0.9212	0.8534	0.8544	0.7057	0.5735	0.9428	0.917
SIDM00238	0.8869	0.8175	0.8869	0.8175	0.8768	0.8113	0.7133	0.5762	0.8869	0.8173
SIDM00815	0.9075	0.8883	0.9064	0.8855	0.5541	0.4659	0.6912	0.5702	0.9106	0.8942
SIDM00048	0.8732	0.8549	0.8732	0.8549	0.5249	0.4471	0.6951	0.5719	0.8742	0.8473
SIDM00231	0.9154	0.8198	0.9154	0.8198	0.6054	0.3376	0.6937	0.5693	0.9203	0.8275
SIDM00232	0.9066	0.8333	0.9066	0.8333	0.8299	0.7356	0.6945	0.5711	0.9054	0.8462
SIDM00819	0.9184	0.8685	0.9184	0.8685	0.5782	0.4106	0.7022	0.5771	0.9172	0.8677

Continued on next page

Table S8 – continued from previous page

Cell line	Ridge		Lasso		CNN		tCNN		BKDRP	
	AUC	AUPR	AUC	AUPR	AUC	AUPR	AUC	AUPR	AUC	AUPR
SIDM00818	0.9102	0.9293	0.9102	0.9293	0.6439	0.6506	0.687	0.5618	0.9112	0.9314
SIDM00820	0.8707	0.8406	0.8707	0.8406	0.5435	0.3978	0.671	0.5485	0.8641	0.8302
SIDM00821	0.9349	0.8778	0.9349	0.8778	0.5291	0.3702	0.7004	0.5803	0.9384	0.8824
SIDM00816	0.8863	0.7469	0.8863	0.7469	0.586	0.3436	0.6954	0.5682	0.8892	0.7575
SIDM00608	0.9101	0.925	0.9101	0.925	0.6717	0.647	0.6816	0.5612	0.9152	0.9304
SIDM00459	0.8845	0.9328	0.887	0.9333	0.6752	0.8112	0.6768	0.5562	0.8883	0.9377
SIDM00193	0.8734	0.5851	0.8734	0.5851	0.4903	0.2181	0.7036	0.5714	0.8734	0.565

Table S9: LOCO performance (AUC and AUPR) of BKDRP and baseline methods across cancer types.

Cancer types	Ridge		Lasso		CNN		tCNN		BKDRP	
	AUC	AUPR	AUC	AUPR	AUC	AUPR	AUC	AUPR	AUC	AUPR
ACC	0.9309	0.9367	0.9309	0.9367	0.7502	0.7258	0.6811	0.6362	0.9309	0.9329
ALL	0.8142	0.8053	0.8005	0.7832	0.5333	0.5333	0.7107	0.6776	0.8824	0.8959
BLCA	0.8877	0.7661	0.8848	0.7669	0.5609	0.3185	0.688	0.4315	0.9029	0.7986
BRCA	0.8126	0.6466	0.8046	0.6189	0.7689	0.6428	0.6447	0.4456	0.843	0.7422
CESC	0.8704	0.7515	0.8509	0.7343	0.5489	0.3309	0.6547	0.399	0.896	0.7767
CLL	0.9459	0.9732	0.9459	0.9732	0.6002	0.721	0.7004	0.7716	0.947	0.973
COREAD	0.8018	0.6641	0.7994	0.6273	0.647	0.4851	0.6748	0.5059	0.8711	0.791
DLBC	0.8302	0.845	0.8186	0.8329	0.607	0.6377	0.6977	0.6711	0.8612	0.8675
ESCA	0.8588	0.7835	0.8626	0.7856	0.646	0.4896	0.6821	0.5109	0.8719	0.7987
GBM	0.8569	0.7432	0.8654	0.7468	0.5306	0.3285	0.6953	0.459	0.8721	0.7703
HNSC	0.809	0.7259	0.8175	0.7304	0.4896	0.3707	0.6511	0.525	0.8424	0.7718
KIRC	0.8512	0.7171	0.8416	0.6987	0.5396	0.3085	0.6547	0.433	0.8654	0.7287
LAML	0.7952	0.8099	0.8005	0.8079	0.5609	0.5761	0.6891	0.659	0.8433	0.8531
LCML	0.8307	0.8532	0.8013	0.8192	0.5469	0.5876	0.7086	0.7081	0.9028	0.9145
LGG	0.8898	0.7859	0.8775	0.7624	0.5369	0.3136	0.7074	0.4665	0.8923	0.7748
LIHC	0.8638	0.6992	0.8585	0.6848	0.5501	0.2616	0.6987	0.3862	0.8903	0.7112
LUAD	0.8391	0.6892	0.8353	0.6857	0.6907	0.4799	0.664	0.4132	0.8488	0.6871
LUSC	0.8328	0.7102	0.831	0.7187	0.6197	0.4489	0.7014	0.5538	0.9216	0.8677
MB	0.8471	0.8226	0.851	0.8243	0.6908	0.6345	0.7399	0.6502	0.9447	0.9325
MESO	0.8642	0.6501	0.8563	0.6393	0.6578	0.3678	0.7527	0.4264	0.8866	0.6767
MM	0.8299	0.7942	0.8267	0.7939	0.5997	0.5296	0.7087	0.6228	0.8844	0.8535
NB	0.8444	0.7879	0.8396	0.7734	0.7657	0.6918	0.7354	0.5909	0.8761	0.8351
OV	0.85	0.7056	0.8389	0.683	0.6615	0.4732	0.6845	0.4482	0.869	0.7339
PAAD	0.8628	0.6417	0.8561	0.6297	0.6685	0.3437	0.6482	0.3249	0.8857	0.7096
PRAD	0.7842	0.5759	0.8294	0.6696	0.3456	0.2105	0.7366	0.4103	0.9037	0.7802
SCLC	0.8391	0.7147	0.8183	0.6702	0.524	0.3308	0.6982	0.4681	0.8671	0.769
SKCM	0.8325	0.7075	0.8281	0.7074	0.5105	0.3391	0.7047	0.4913	0.8354	0.7375
STAD	0.8286	0.7495	0.809	0.7215	0.6547	0.5171	0.7285	0.5266	0.8666	0.7868
THCA	0.8684	0.7291	0.8517	0.7282	0.787	0.6215	0.7186	0.4749	0.9128	0.8238
UCEC	0.8542	0.6759	0.8821	0.7543	0.5942	0.3296	0.7723	0.4481	0.9182	0.7903

Table S10 shows the statistical comparison using the Wilcoxon signed-rank test of BKDRP against the baseline models for three evaluation settings: LOCLO, LODO, and LOCO. In the table W represents the Wilcoxon test statistic, and p represents the corresponding p-value. If $p < 0.05$, i.e., p-value is less than the significant cut-off 5%, then the result is statistically significant.

Table S10: Statistical comparison (Wilcoxon signed-rank test) of BKDRP against baseline models across LOCLO, LODO, and LOCO.

Comparison	LOCLO				LODO				LOCO			
	AUC		AUPR		AUC		AUPR		AUC		AUPR	
	W	p	W	p	W	p	W	p	W	p	W	p
BKDRP vs Ridge	85045.5	0	121543	0.0174	347	0	684	0.0045	1	0	10	0
BKDRP vs Lasso	86119	0	123449	0.0406	987	0.3424	761	0.0182	1	0	4	0
BKDRP vs tCNN	13	0	666	0	22	0	64	0	0	0	0	0
BKDRP vs CNN	9	0	35	0	60	0	4	0	0	0	0	0

Table S11: Ablation study of BKDRP and Performance comparison with MLP.

Experiment	Run	Accuracy	AUC	AUPR
Only gene expression + BKDRP	1	0.8404	0.9121	0.8545
	2	0.8379	0.9111	0.8521
	3	0.8388	0.9118	0.8532
	4	0.8399	0.9118	0.8537
	5	0.8407	0.9119	0.8537
w/o copy number variation + BKDRP	1	0.8396	0.9134	0.8568
	2	0.843	0.9135	0.8565
	3	0.8395	0.9133	0.8562
	4	0.8394	0.9131	0.8554
	5	0.8416	0.9135	0.8563
w/o mutation + BKDRP	1	0.8399	0.9135	0.8564
	2	0.8402	0.9132	0.8564
	3	0.8421	0.9136	0.8572
	4	0.8412	0.9133	0.856
	5	0.8424	0.9137	0.8567
w/o protein + BKDRP	1	0.8406	0.9131	0.8553
	2	0.843	0.9135	0.8564
	3	0.8406	0.9135	0.8562
	4	0.8422	0.9132	0.8566
	5	0.8418	0.9134	0.8563
All omics + BKDRP	1	0.8421	0.9131	0.8557
	2	0.8426	0.9139	0.8566
	3	0.84	0.9134	0.8563
	4	0.8419	0.9142	0.8575
	5	0.8422	0.9138	0.8562
All omics + MLP	1	0.6669	0.6511	0.4691
	2	0.666	0.6522	0.4684
	3	0.6686	0.653	0.4705
	4	0.6672	0.6528	0.4696
	5	0.6681	0.6535	0.4696
All omics + fingerprints + MLP	1	0.8213	0.8462	0.8056
	2	0.8202	0.8408	0.8005
	3	0.8237	0.85	0.806
	4	0.8224	0.8385	0.799
	5	0.8217	0.8469	0.8051
